# The *sst1* Resistance Locus Regulates Evasion of Type I Interferon Signaling by *Chlamydia pneumoniae* as a Disease Tolerance Mechanism

**DOI:** 10.1371/journal.ppat.1003569

**Published:** 2013-08-29

**Authors:** Xianbao He, Robert Berland, Samrawit Mekasha, Thomas G. Christensen, Joseph Alroy, Igor Kramnik, Robin R. Ingalls

**Affiliations:** 1 Section of Infectious Diseases, Boston University School of Medicine and Boston Medical Center, Boston, Massachusetts, United States of America; 2 National Emerging Infectious Diseases Laboratories Institute and Pulmonary Center, Boston University School of Medicine, Boston, Massachusetts, United States of America; 3 Department of Pathology and Laboratory Medicine, Boston University School of Medicine, Boston, Massachusetts, United States of America; 4 Department of Pathology, Tufts University School of Medicine and Tufts Medical Center, Boston, Massachusetts, United States of America; Duke University, United States of America

## Abstract

The *sst1*, “*supersusceptibility to tuberculosis*,” locus has previously been shown to be a genetic determinant of host resistance to infection with the intracellular pathogen, *Mycobacterium tuberculosis*. *Chlamydia pneumoniae* is an obligate intracellular bacterium associated with community acquired pneumonia, and chronic infection with *C. pneumoniae* has been linked to asthma and atherosclerosis. *C. pneumoniae* is a highly adapted pathogen that can productively infect macrophages and inhibit host cell apoptosis. Here we examined the role of *sst1* in regulating the host response to infection with *C. pneumoniae*. Although mice carrying the *sst1* susceptible (*sst1^S^*) locus were not impaired in their ability to clear the acute infection, they were dramatically less tolerant of the induced immune response, displaying higher clinical scores, more severe lung inflammation, exaggerated macrophage and neutrophil influx, and the development of fibrosis compared to wild type mice. This correlated with increased activated caspase-3 in the lungs of infected *sst1^S^* mice. Infection of *sst1^S^* macrophages with *C. pneumoniae* resulted in a shift in the secreted cytokine profile towards enhanced production of interferon-β and interleukin-10, and induced apoptotic cell death, which was dependent on secretion of interferon-β. Intriguingly macrophages from the *sst1^S^* mice failed to support normal chlamydial growth, resulting in arrested development and failure of the organism to complete its infectious cycle. We conclude that the *sst1* locus regulates a shared macrophage-mediated innate defense mechanism against diverse intracellular bacterial pathogens. Its susceptibility allele leads to upregulation of type I interferon pathway, which, in the context of *C. pneumoniae*, results in decreased tolerance, but not resistance, to the infection. Further dissection of the relationship between type I interferons and host tolerance during infection with intracellular pathogens may provide identification of biomarkers and novel therapeutic targets.

## Author Summary

*Chlamydia pneumoniae* is a highly adapted intracellular pathogen and a common cause of atypical, community acquired pneumonia. It has also been suggested as a trigger or promoter of asthma and atherosclerosis. In this study, we examined the role of a genetic locus on mouse chromosome 1 that has been associated with susceptibility to another intracellular pathogen, *Mycobacterium tuberculosis*, in the pathogenesis of respiratory infections secondary to *Chlamydia pneumoniae*. We have determined that a variant at this locus, known as *sst1* and associated with destructive pulmonary tuberculosis, makes mice dramatically more sensitive *in vivo* to the inflammatory changes following respiratory infection with *C. pneumoniae*. This appears to arise from activation of type I interferons and apoptotic cell death, two signaling pathways that are normally silent during productive *C. pneumoniae* infection. Despite a noted inability of *sst1* susceptible macrophages to support chlamydial development, exuberant lung tissue damage resulted in overall more severe disease *in vivo*. We conclude the *sst1*-mediated control of lung tissue damage is an important determinant of the genetic susceptibility of a given host to a number of diverse intracellular bacterial pathogens, which may provide predictors of outcomes to infectious diseases as well as possible target for novel therapeutics.

## Introduction

The susceptibility of a host to infection, as well as the immunologic response to a given infection, is heterogeneous within a population. While epidemiologic studies may suggest inherited factors can influence immunity to infectious diseases, it is clear that resistance to infection is a complex, multifactorial genetic trait in which many genetic polymorphisms contribute to the phenotype. Thus, the unique host-pathogen interaction of an individual that determines the outcome of infection is under multigenic control. Inbred strains of mice are well suited for studying the genetic control of infectious diseases as gene effects may have become fixed in inbred, recombinant inbred, and recombinant congenic strains of mice (reviewed in [1], [2]). For example, using an experimental mouse model for *Mycobacterium tuberculosis* infection in C57BL/6 mice (inherently resistant to *M. tuberculosis* infection) and C3HeB/FeJ mice (inherently susceptible to *M. tuberculosis*), we previously identified a locus on chromosome 1 that has a significant effect on the development of disease following *M. tuberculosis* infection, particularly within the lung. We designated this as the *sst1* locus, for “super susceptibility to tuberculosis”, and demonstrated that the presence of the C57BL/6 *sst1* locus imparts resistance to *M. tuberculosis* infection when expressed on the C3HeB/FeJ background [3], while the C3HeB/FeJ *sst1* locus imparts susceptibility to *M. tuberculosis* when expressed on the C57BL/6 background [4]. Thus, C57BL/6 mice are deemed “resistant” at the *sst1* locus (or *sst1^R^*), while C3HeB/FeJ mice are “susceptible” (or *sst1^S^*).

The *sst1* locus appears to participate in control of intracellular multiplication of virulent *M. tuberculosis* within the macrophage, and has an effect on the infected macrophages' mechanism of cell death. However, the effects of the *sst1* locus are not specific to *M. tuberculosis* as it has also been shown to be important in infections due to another bacterial species with a significant intracellular phase, *Listeria monocytogenes* [5]. Subsequent studies identified a candidate gene, *Intracellular Pathogen Resistance 1 (Ipr1)*, within the *sst1* locus as playing a role in controlling innate immunity and cell death in response to both *M. tuberculosis* and *Listeria* [6]. The closest human homologue to *Ipr1* is *Sp110*, and mutations in *Sp110* have been associated with hepatic veno-occlusive disease with immunodeficiency (VODI) [7], [8]; however, the role of *Sp110* in human tuberculosis is still being debated [9], [10], [11]. The identification of additional pathogens that are controlled by genes within the *sst1* locus may shed light on the *sst1/Sp110*-mediated mechanism of innate host resistance to divergent intracellular pathogens, as well as their shared pathogenesis and immune evasion strategies.

*Chlamydia pneumoniae* is an obligate intracellular pathogen and the etiologic agent of atypical, community acquired pneumonia. While respiratory infection is often subclinical or mildly symptomatic, chronic infection with *C. pneumoniae* has been suggested as a trigger or promoter of a number of inflammatory conditions, including asthma (reviewed in [12], [13]), chronic obstructive pulmonary disease (COPD) [14], and atherosclerosis (reviewed in [15]). The mechanism by which *C. pneumoniae* can disseminate from the lung remains unproven, but one leading model suggests that replication within lung macrophages provides a unique niche for transport to distant sites, such as chronic vascular lesions (reviewed in [15]), and case reports have identified *C. pneumoniae* within atherosclerotic lesions [16], [17].

Like all Chlamydia species, *C. pneumoniae* has a unique dimorphic developmental cycle that sets it apart from other bacterial species. There are two major developmental forms that are recognized: the infectious, but metabolically inert form, known as the elementary body (EB); and the intracellular, replicative form known as the reticulate body (RB). The developmental cycle begins with cellular attachment and entry of the EB, which then converts into the RB form and begins to replicate by binary fission. These RBs grow and divide within a unique membrane-bound cellular inclusion, and at some point mid to late in the infectious process, RBs differentiate back to EBs, which are eventually released coincident with cell lysis into the extracellular space, around 40 to 72 hours post infection, depending on the species of *Chlamydia*. Then, a new round of infection and development begins. While this acute or lytic lifestyle has been extensively studied, *Chlamydia* species can also enter a less-understood persistent state, defined as the presence of large, irregular, non-cultivatable developmental forms that fail to mature; these have been referred to as aberrant bodies or aberrant reticulate bodies (reviewed in [18]). *In vitro*, a number of seemingly unrelated triggers have been shown to induce persistence, including antibiotic exposure [19], nutritional deprivation (*e.g.*, amino acid deficiency [20], iron limitation [21]), and exposure to interferon (IFN)-γ, which is known to upregulate cellular indoleamine 2,3-dioxygenase (IDO) and thus deplete intracellular tryptophan stores [22]. The role of chlamydial persistence and these aberrant developmental forms in the pathogenesis of any of the clinical syndromes associated with chlamydia infections remains unclear.

Given the limited innate immune evasion strategies of an obligate intracellular pathogen, and the similarities between the lifestyle of *Mycobacteria* and *Chlamydia*, we examined the host-pathogen interaction between *C. pneumoniae* and eukaryotic macrophages in the context of the *sst1* locus with the goal of identifying key pathways involved in pathogenesis. We observed that the *sst1* locus regulates the innate immune response of macrophages to *C. pneumoniae*, altering the cytokine balance towards IL-10 and IFN-β, and overriding the chlamydial anti-apoptotic program, which resulted in arrested bacterial development. Paradoxically, this translated *in vivo* into a more severe clinical course in a pneumonia model, with enhanced apoptosis and fibrosis within lung tissue of infected mice, in spite of their ability to control bacterial replication. Our data demonstrate that *sst1* is a key host susceptibility locus for intracellular pathogens, regulating inflammation and cell death in response to infection.

## Results

### The *sst1* locus controls the innate immune response to lung infection with *C. pneumoniae*

To examine the effect of the *sst1* locus on susceptibility of mice to *C. pneumoniae* (Cp) infection, we compared standard inbred C57BL/6 (B6) mice, which are resistant at the *sst1* locus, and *sst1* susceptible congenic mice, B6.C3H-*sst1*. The B6.C3H-*sst1* strain is 99.5% genetically identical to the resistant B6 strain with the exception of a 12-cM interval on mouse chromosome 1 encompassing the *sst1* locus, which has been introgressed from the susceptible C3HeB/FeJ strain [23]. Mice were infected with *C. pneumoniae* as described in the Methods section, and their clinical state was scored on a scale of 0 to 4, with a score of 0 for mice that did not appear clinically ill and a score of 4 for mice that were moribund and required euthanasia. As shown in **Table 1**, B6.C3H-*sst1* mice overall displayed a higher clinical score compared to wild type B6 mice, with a statistically significant difference between the mean scores of 1.64 in B6 mice vs. 2.60 in the B6.C3H-*sst1* mice (p = 0.0014). Moreover, 15 out of 25 mice in the B6.C3H-*sst1* group reached a score of 3–4, corresponding to severe illness or moribund state, compared to only 5 out of 25 mice in the B6 group (**Table S1**). Although both groups experienced weight loss as a result of infection, there was no statistically significant difference between the B6 and B6.C3H-*sst1* mice over the first week of infection (**Figure S1A**). Quantitative culture of lung homogenates similarly revealed no statistically significant differences in clearance of the bacterial load in the lungs between the B6 and B6.C3H-*sst1* mice (**Figure S1B**), nor was there any difference in dissemination of bacteria to the spleen (data not shown). This suggested clinically more severe disease in the infected B6.C3H-*sst1* mice that could not be explained by impaired control of bacterial replication.

**Table 1:** Clinical scores.

Description	Severity grade	B6 N = 25	B6.C3H-*sst1* N = 25
Moribund	4	0	5
Severe illness	3	5	10
Mild-moderate illness	2	8	6
Subtle illness	1	10	3
Healthy	0	2	1
	Median	2	3
	Mean (+/−SEM)	1.64 (+/−0.18) **	2.60 (+/−0.22) **

Clinical scores were determined on day 6 post-infection, and reported above as the number of mice that fell into each severity group. Median and mean +/− SEM are shown. Significance: ** p = 0.0014 by two-tailed unpaired t-test. N = 25 mice per genotype, pooled from two independent experiments.

**Figure 1:**
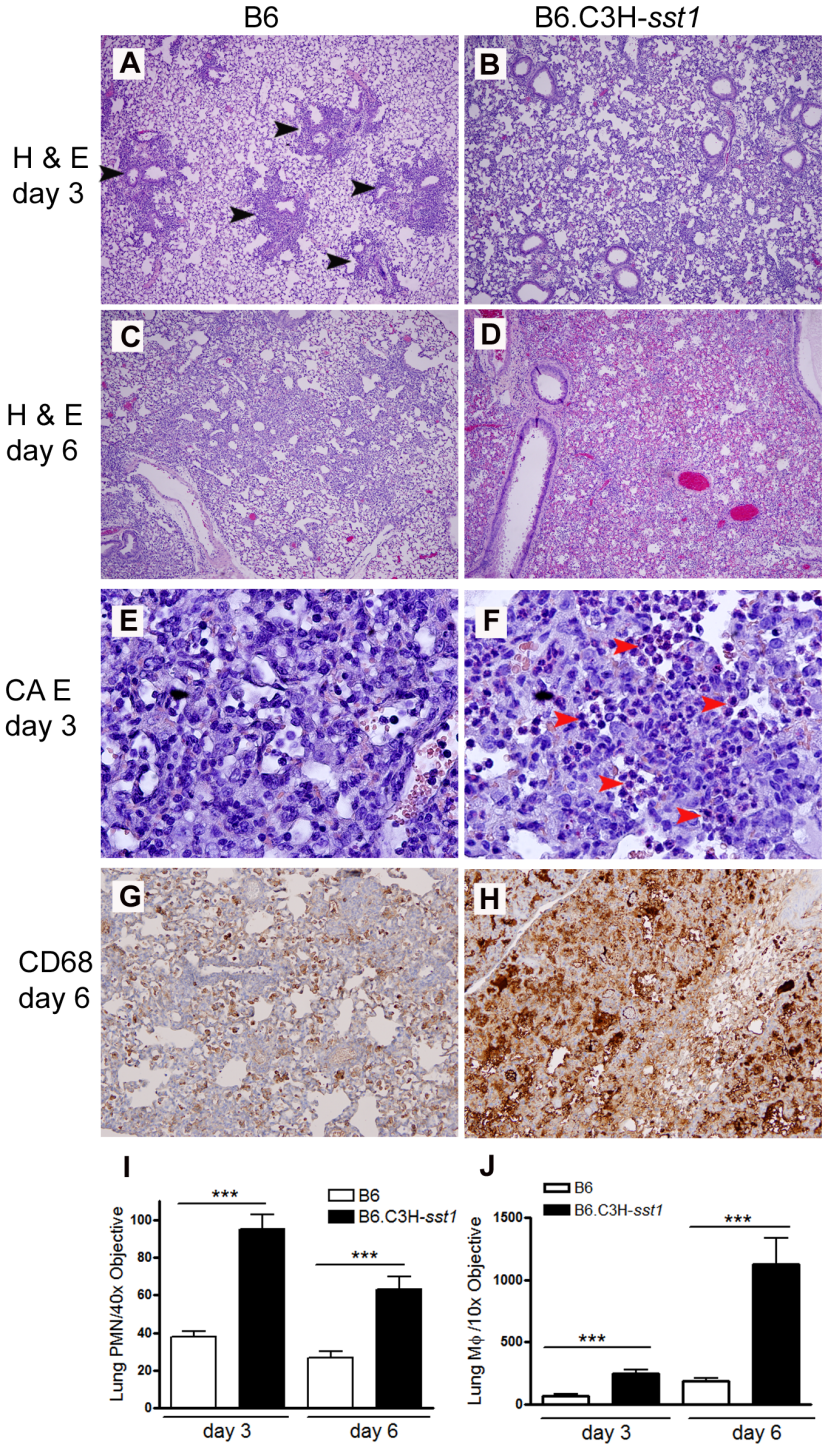
*C. pneumoniae* infected B6.C3H-*sst1* mice develop diffuse lung inflammation compared to B6 mice. C57BL/6 (B6) or B6.C3H-*sst1* congenic mice were infected with *C. pneumoniae* as described in the Methods. At day 3 and day 6 post infection, mice were euthanized and lungs removed for histologic analysis. A–D: routine H & E staining for day 3 (A–B) and day 6 (C–D). Black arrow heads indicate areas of patchy inflammatory infiltrate. E–F: CAE staining for detection of neutrophils, indicated by red arrow heads. G–H: immunohistochemistry for the macrophage marker CD68, shown by brown staining. Graphs at the bottom represent quantified data from (I) neutrophil images E–F and (J) macrophage images G–H. Original magnification: A–D, 40×; E–F, 400×; G–H, 100×. Shown above are images from one of four infected mice from each genetic background. The result is a representative of two independent experiments. ***, p≤0.001.

When histological sections of the lung were examined from infected mice, we observed qualitative differences in the inflammatory response. Infected B6 mice displayed patchy, focal areas of inflammation, while the B6.C3H-*sst1* mice developed more diffuse, extensive inflammation. This was true on both day 3 (**Figure 1 A–B**) and day 6 (**Figure 1 C–D**) post-infection, suggesting a defective ability of the B6.C3H-*sst1* mice to regulate inflammation in the lungs during the period of bacterial replication. We also observed an exaggerated influx of both PMNs (**Figure 1 E–F**) and macrophages (**Figure 1 G–H**) in the lungs of B6.C3H-*sst1* mice compared to B6 mice histologically, which was confirmed when the images were quantified (**Figure 1 I–J**). Furthermore, we observed increased immunostaining for vimentin in the lung parenchyma of the B6.C3H-*sst1* mice as early as day 3, along with enhanced collagen deposition consistent with fibrosis, as detected by Masson's trichrome staining, and increased staining for thyroid transcription factor-1 (TTF-1), a nuclear protein expressed in type II pneumocytes (**Figure S2**). These data are consistent with increased tissue damage and repair in response to infection in the B6.C3H-*sst1* mice.

**Figure 2:**
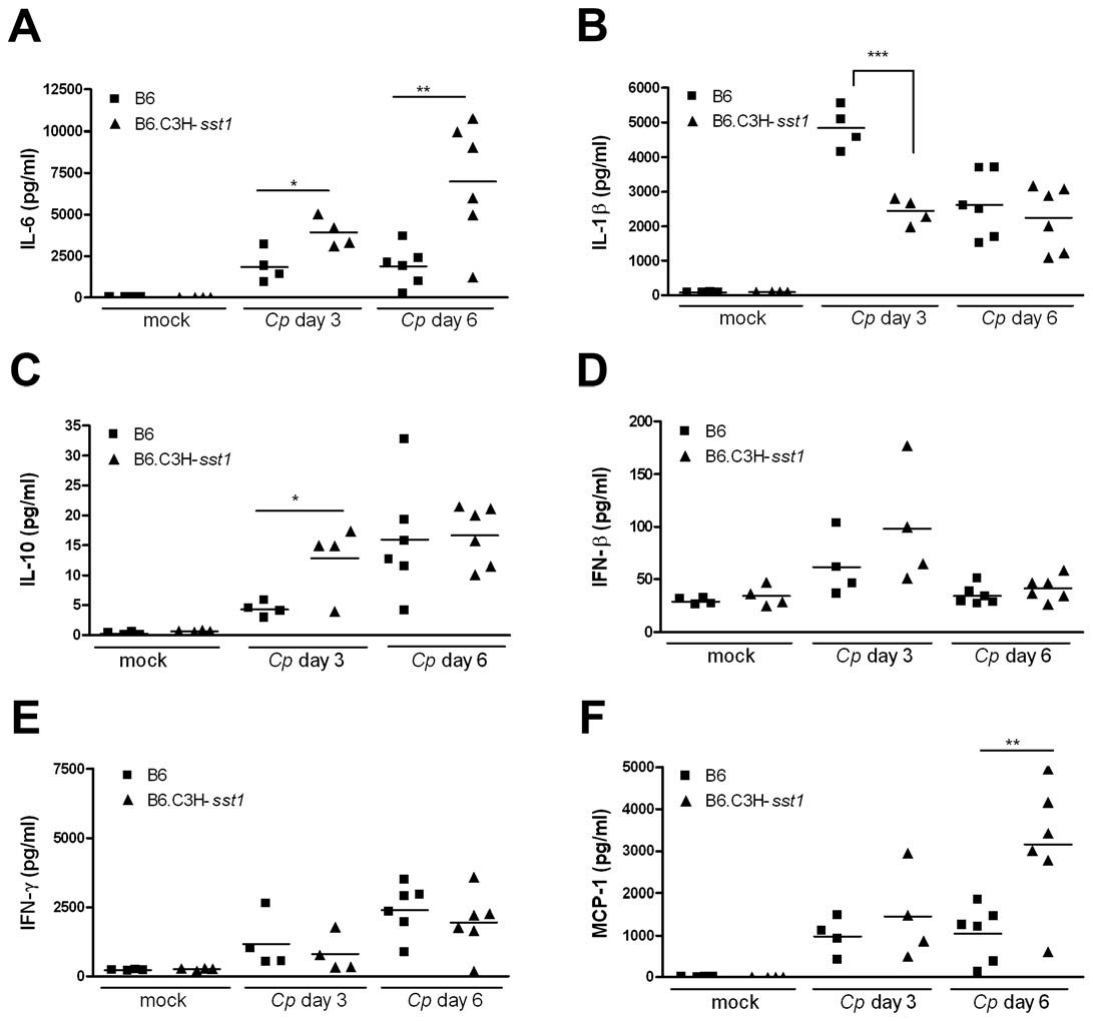
Cytokine production from the lung homogenates of *C. pneumoniae* infected mice. C57BL/6 (B6) or B6.C3H-*sst1* congenic mice were infected with *C. pneumoniae* (Cp) as described in the Methods. At day 3 and day 6 post infection, mice were euthanized and lung homogenates prepared for detection of a panel of inflammatory cytokines and chemokines. Shown above are a subset of the cytokines assayed: (A) IL-6, (B) IL-1β, (C) IL-10, (D) IFN-β, (E) IFN-γ and (F) MCP-1. Each data point represents one mouse, and the horizontal bar represents the mean. Significance: *, p<0.05; **, p≤0.01; ***, p≤0.001 infected C57BL/6 vs. B6.C3H-*sst1* mice. The result is representative of two independent experiments.

When we examined lung homogenates for the presence of inflammatory mediators (**Figure 2**), we observed a bias towards an anti-inflammatory response in the B6.C3H-*sst1* mice, with exaggerated IL-10, a classic anti-inflammatory cytokine, and IL-6, which can have both pro- and anti-inflammatory activity [24]. Although there was a trend towards higher IFN-β in the infected B6.C3H-*sst1* mice, it did not reach statistical significance. The B6.C3H-*sst1* mice also displayed higher levels of MCP-1 (also known as CCL-2) in the lungs, consistent with the exaggerated influx of monocytes, while the IFN-γ response was elevated but not significantly different between the two infected mouse strains. Together, these data demonstrate a dysregulated inflammatory and fibrogenic response in Cp-infected B6.C3H-*sst1* mice, which carry the *sst1* susceptible locus.

### The *sst1* locus regulates IFN-β and IL-10 production in *C. pneumoniae*-infected macrophages

**Figure 3:**
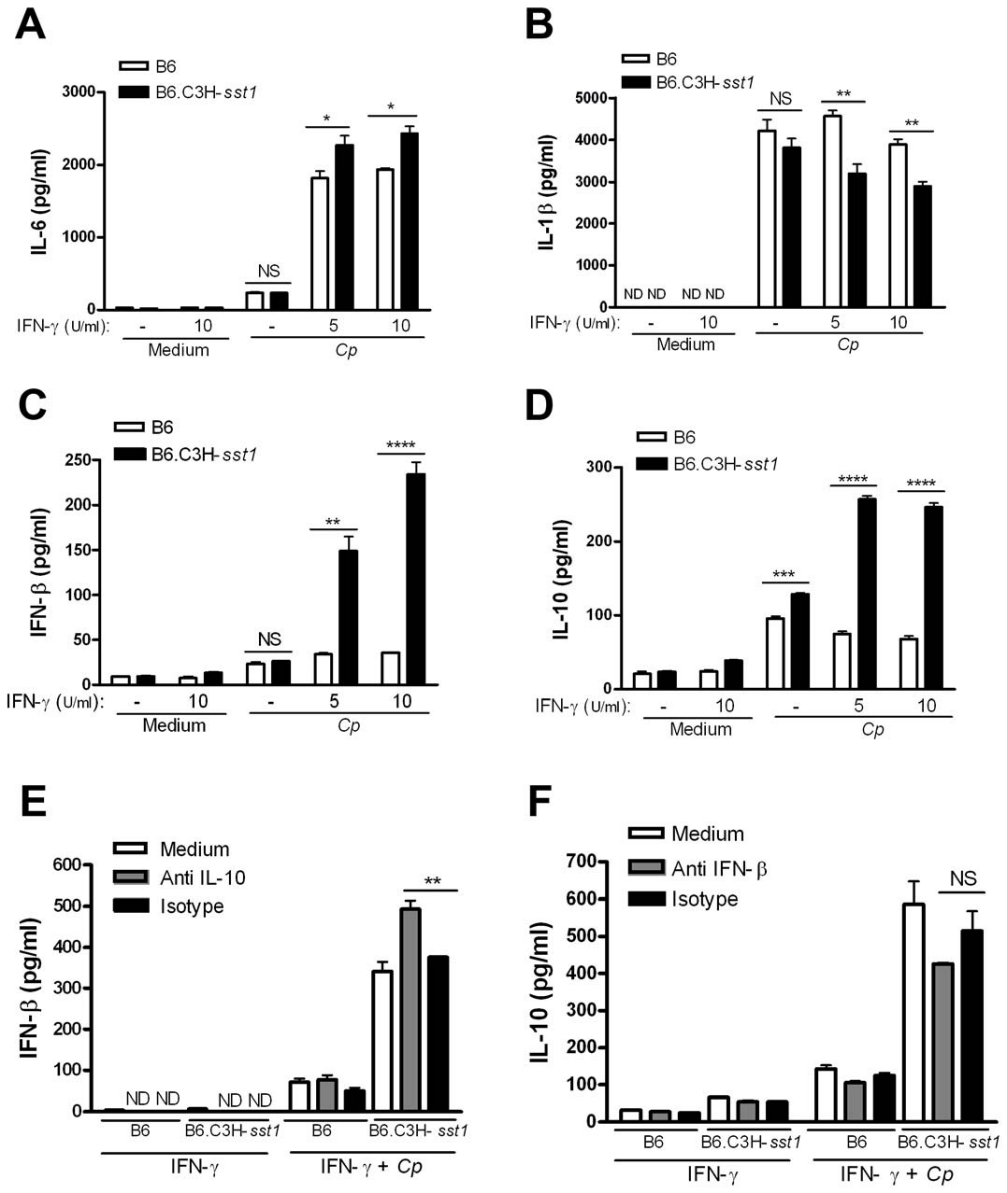
*In vitro* cytokine production from *C. pneumoniae* infected BMDM. BMDMs were prepared from C57BL/6 (B6) or B6.C3H-*sst1* congenic mice, as described in the Methods. Cells were infected with *C. pneumoniae* at an MOI of 3∶1, and supernatant harvested at 24 hpi for assay of the indicated cytokines. (A–D) In some cases, cells were pretreated with IFN-γ for 30 min at the indicated concentrations prior to infection. (E–F) For antibody neutralization, cells were pretreated with indicated neutralizing antibody (anti-IL-10 or anti-IFN-β) or isotype control at a concentration of 250 ng/ml prior to infection or IFN-γ treatment (10 U/ml). Data is shown as the mean ± SEM from triplicate wells. Significance: NS, No significant difference; *, p<0.05; **, p≤0.01; ***, p≤0.001; ****, p≤0.0001. The results are representative of at least 3 independent experiments.

Type I IFNs are known to play a critical role in the immune response to viruses and intracellular bacteria, but they have also been shown to possess anti-inflammatory effects as well (reviewed in [25], [26]). For example, IFN-β was recently reported to inhibit IL-1β production and inflammasome activation [27]. To better understand the *sst1* regulated anti-inflammatory cytokine response, we prepared bone marrow derived macrophages (BMDM) from B6.C3H-*sst1* mice and wild type B6 mice, and infected them *in vitro* with Cp in the presence or absence of IFN-γ priming. As shown in **Figure 3 A–D**, we observed significantly higher levels of IL-6 and IL-10, with lower IL-1β production, in IFN-γ primed B6.C3H-*sst1* macrophages compared to wild type B6 macrophages, similar to what we observed *in vivo*. The differences were not as significant in the unprimed macrophages. The most striking effect, however, was observed in type I interferon response. As shown in **Figure 3C**, wild type B6 BMDM secrete barely detectable levels of IFN-β in response to Cp infection regardless of IFN-γ priming. In contrast, Cp infection of IFN-γ primed B6.C3H-*sst1* BMDM resulted in a three to fourfold increase in IFN-β induction compared to B6 BMDM (**Figure 3C**).

It has been shown that type I IFN induction is required for LPS-induced IL-10 production [28]. To investigate whether IFN-β and IL-10 induction was linked in the response of B6.C3H-*sst1* BMDM to Cp infection, we performed IFN-β and IL-10 neutralization assays. As shown in **Figure 3 E–F**, neutralization of IL-10 significantly enhanced IFN-β production while neutralization of IFN-β resulted in a modest but not statistically significant reduction in IL-10 production, which might reflect residual signaling by other type I IFNs. Thus, while IFN-β and other type I IFNs may be upstream of IL-10, IL-10 itself appears to negatively regulate IFN-β in B6.C3H-*sst1* BMDM.

**Figure 4:**
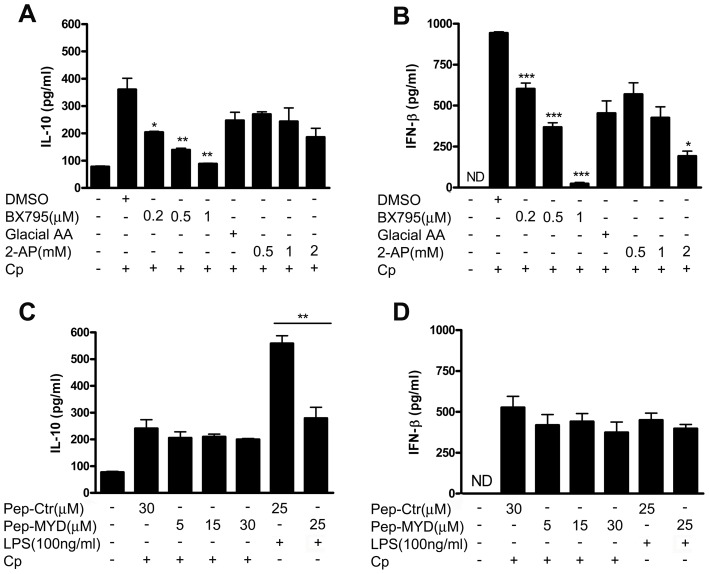
*C. pneumoniae* induces IFN-β and IL-10 production in BMDM from B6.C3H-*sst1* via TBK1/IKKξ dependent pathway and is independent of MyD88. BMDM were prepared from B6.C3H-*sst1* mice as described in the Methods. Prior to infection with *C. pneumoniae* (Cp) or treatment with LPS, cells were pretreated with IFN-γ (5 U/ml) and the following inhibitors for 30 min at the indicated concentrations; supernatant was harvested at 24 hpi for assay of IL-10 and IFN-β. A–B: TBK1/IKKξ inhibitor BX795 or vehicle control (DMSO); or the PKR inhibitor 2-aminopurine (2-AP) or vehicle control PBS∶Glacial acetic acid at a ratio of 200∶1 (Glacial AA). C–D: MYD88 inhibitor Pepinh-MYD (Pep-MYD), or Pepinh-control (Pep-Ctr) Significance: *, p≤0.05; **, p≤0.01; ***, p≤0.001 compared to corresponding control. The results are representative of 2 independent experiments.

To further examine the mechanism by which Cp infection of B6.C3H-*sst1* BMDM resulted in the upregulation of IL-10 and IFN-β, we examined the effect of specific pathway inhibitors on this response. Two related IκB kinase homologues, IκB kinase-**ξ** (IKK**ξ**) [29], [30] and TANK-binding kinase 1 (TBK1) [31], [32], are essential components of the IRF3 signaling pathway upstream of type I interferon induction [33]. We observed that the pharmacological inhibitor, BX795, shown to be a potent inhibitor of IKK**ξ** and TBK1 [34], was capable of inhibiting Cp-induced secretion of both IL-10 and IFN-β to background levels in B6.C3H-*sst1* BMDM in a dose-dependent manner (**Figure 4 A–B**). Similarly, 2-aminopurine (2-AP), a potent inhibitor of double-stranded RNA (dsRNA)-activated protein kinase (PKR) [35] that has also been shown to inhibit nuclear translocation of phosphorylated-IRF-3 [36], had a modest effect on IFN-β but only at the highest concentration used without significantly altering IL-10. In contrast, inhibition of the TLR adaptor MyD88, which is shared by all the TLRs except TLR3, had no effect on IL-10 or IFN-β induction in response to Cp (**Figure 4 C–D**). These data suggest that induction of type I IFNs in response to Cp infection of the B6.C3H-*sst1* BMDM occurs in an IRF3 dependent manner, upstream of IKK**ξ** and TBK1, but independent of MyD88. Partial dependence on PKR suggests involvement of a cytosolic receptor although we cannot rule out involvement of endosomal TLR3.

### The *sst1* locus regulates chlamydial development in macrophages

**Figure 5:**
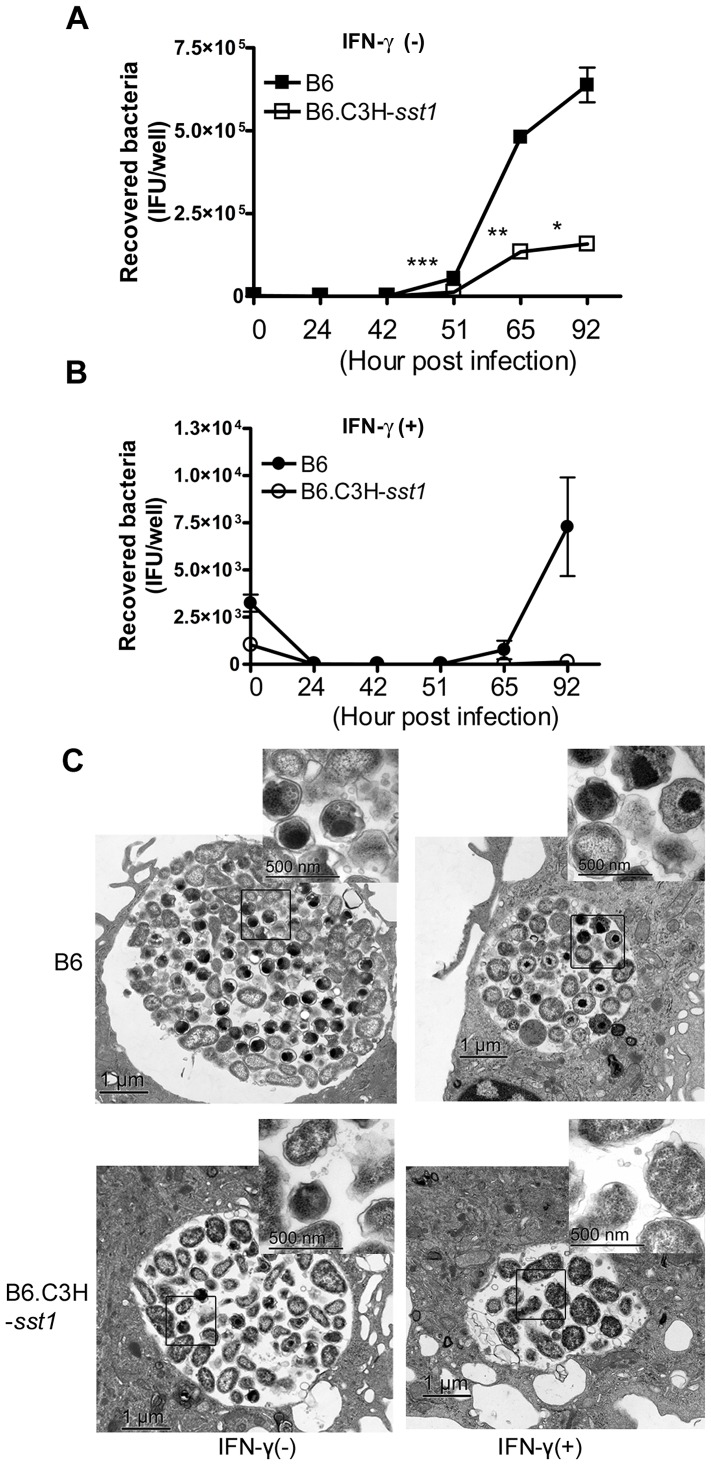
*In vitro C. pneumoniae* growth. BMDMs prepared from C57BL/6 (B6) or B6.C3H-*sst1* mice were infected with *C. pneumoniae* at an MOI of 3∶1, in the absence or presence of IFN-γ (5 U/ml). A–B: Cells were lysed at the indicated time points and recovered EBs were quantitatively cultured, as described in the Methods. Each time point was run in triplicate and data is reported as the mean recovered bacteria per well ± SEM. C: Cells were harvested at 69 hpi and processed for electron microscopy, as described in the Methods. Shown above are representative images from triplicate infected wells. Original magnification: 20,000×, scale bars: 1 µm. High magnification images (upper right inset of each panel): 80,000×, scale bars: 500 nm.

Chlamydiae have a unique dimorphic developmental cycle, with vegetative EBs converting to replicative RBs, and back again. In addition, a third developmental form is recognized, the “aberrant body”, which appears to be an arrested RB which takes on an irregular shape and stops dividing. A number of triggers have been shown to induce the production of aberrant bodies, including IFN-γ, iron or tryptophan starvation, and exposure to penicillins (reviewed in [18]). In order to determine if the *sst1* locus had any impact on Cp replication or development within macrophages, we infected BMDM from B6 and B6.C3H-*sst1* mice with Cp in the absence or presence of IFN-γ, and quantified the burst of recovered EBs over time. As shown in **Figure 5A**, we observed significantly reduced recovery of EBs from Cp grown in B6.C3H-*sst1* BMDM compared to B6 BMDM. Furthermore, while IFN-γ priming reduced the recovery of viable organisms from B6 BMDM by over 1 log, in the case of B6.C3H-*sst1* BMDM we were unable to recover any viable EBs at the end of the culture period (**Figure 5B**). Similarly, we observed significantly fewer inclusions in Cp-infected B6.C3H-*sst1* BMDM compared to wild type B6 BMDM (**Figure S3A**). This defect in Cp development in macrophages was confirmed by electron microscopy. As shown in **Figure 5C**, Cp development within B6.C3H-*sst1* BMDM resulted in smaller inclusions with fewer intracellular bacteria and more aberrant appearing forms, compared to B6 BMDM. Under the stress of IFN-γ priming, this difference was even more exaggerated, and the abnormal inclusions and chlamydial forms observed was quite striking. In fact, we were able to identify just 3–4 intact inclusions in the monolayer of infected, primed B6.C3H-*sst1* macrophages, and the ones that were found were small, surrounded by an irregular membrane with enhanced membrane blebbing, and contained no morphologically normal-appearing forms within them (**Figure 5C**, bottom right). The mechanism behind this impaired development was not clear, and we found no evidence that steady-state mRNA or protein levels of IDO differed between the B6 and B6.C3H-*sst1* macrophages, nor did we observe any recovery of organisms when primed B6.C3H-*sst1* macrophages were supplemented with tryptophan (data not shown). Moreover, in contrast to what we observed with BMDM, lung fibroblasts derived from the B6.C3H-*sst1* mice had no defect in their ability to support chlamydial development (**Figure S3**), nor did they have any alteration in cytokine induction (**Figure S4**). Thus, the phenotype appears to be specific to macrophages or macrophage-like cell types.

### Enhanced IFN-β secretion leads to increased cell death and decreased *Cp* replication in macrophages from B6.C3H-sst1 mice carrying the *sst1^S^* locus

**Figure 6:**
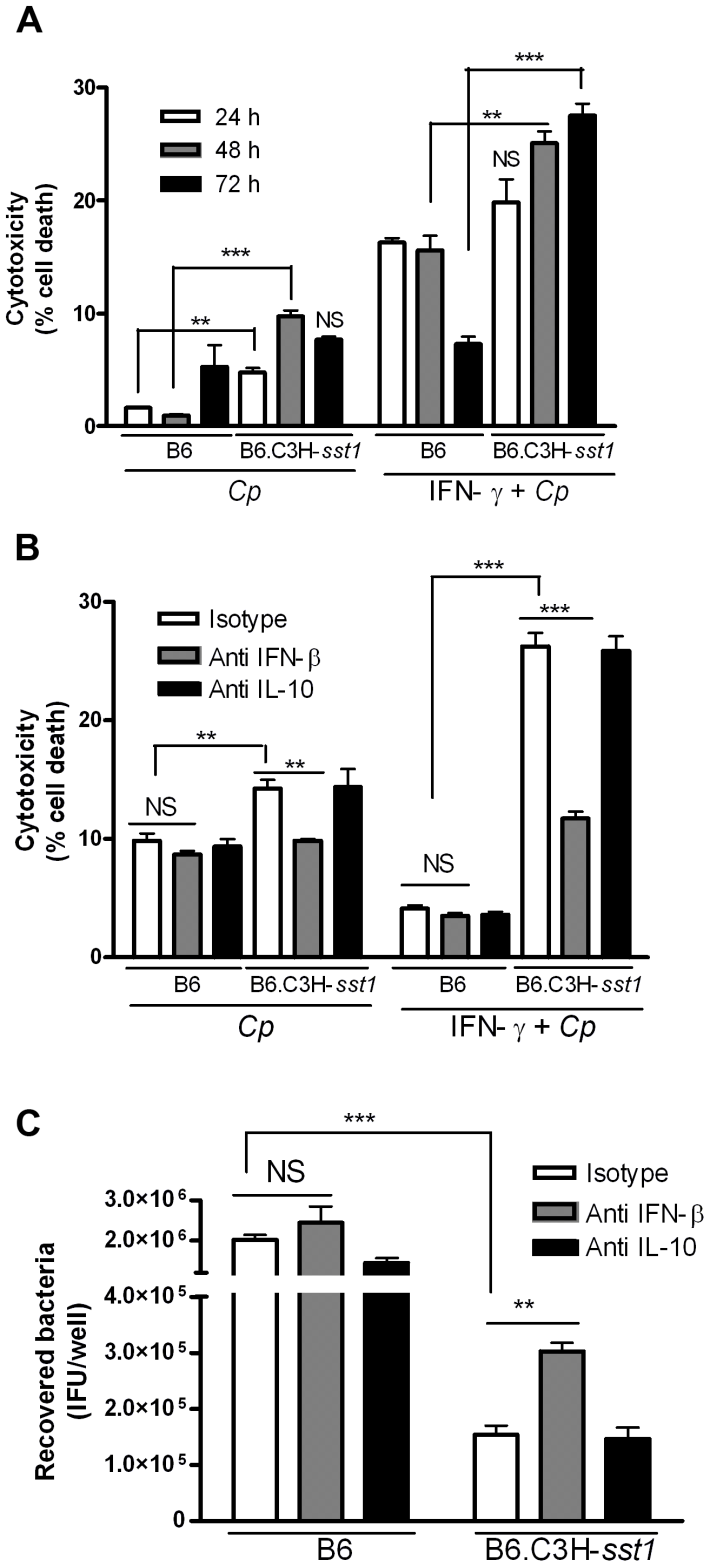
Neutralization of IFN-β protects cells from death and enhances *C. pneumoniae* replication. BMDMs prepared from C57BL/6 (B6) or B6.C3H-*sst1* mice were infected with *C. pneumoniae* (MOI 3∶1) in the presence or absence of IFN-γ (5 U/ml). The ratio of dead cells to total cell number was calculated using propidium iodide and Hoechst staining, as described in the Methods section, and is reported as the percent cytotoxicity. Where indicated, antibody neutralization was carried out using neutralizing antibody against IL-10 or IFN-β, or isotype control at a concentration of 250 ng/ml prior to infection or IFN-γ treatment. A: Cytotoxicity was determined over time at 24, 48 and 72 hpi in Cp infected BMDM, and is reported as percent cell death. B: Effect of IL-10 and IFN-β neutralization on Cp-induced cytotoxicity at 72 hpi. C: Effect of IL-10 and IFN-β neutralization on Cp growth. Infected BMDM were lysed at 69 hpi, and recovered EBs were quantitatively cultured, as described in the Methods. All data shown above represents the mean ± SEM from triplicate wells, and is representative of at least two independent experiments. Significance: *, p<0.05; **, p≤0.01; ***, p≤0.001.

**Figure 7:**
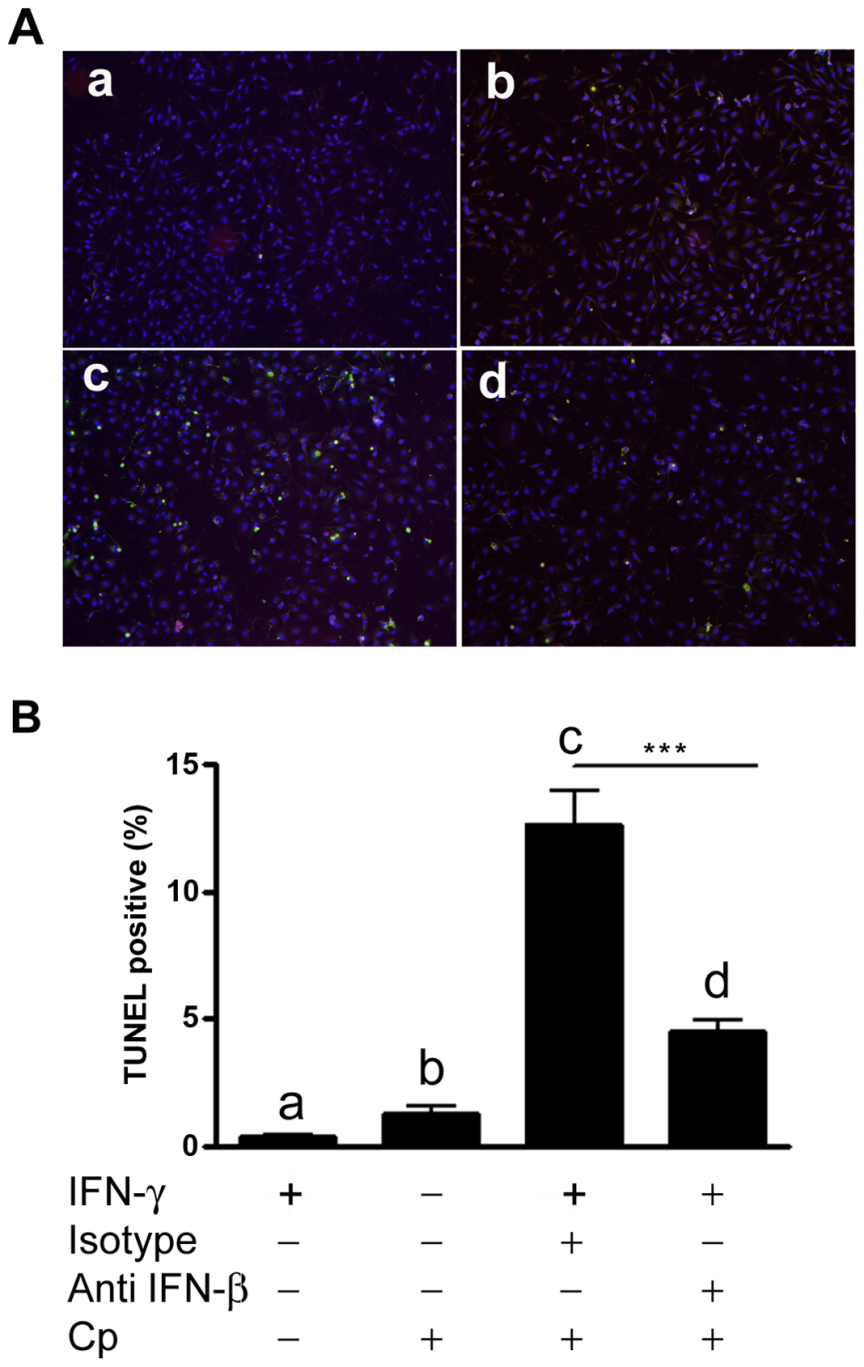
Neutralization of IFN-β protects cells from apoptosis. BMDM were prepared from B6.C3H-*sst1* mice. Where indicated (panel a, c and d), cells were primed with IFN-γ (10 U/ml). Cells were incubated with neutralizing Ab against IFN-β (panel d) or isotype control (panel c) at a final concentration of 250 ng/ml 6 hr prior to infection with Cp (MOI 3∶1). At 10 hpi, cells were fixed and stained using the TUNEL assay. A: fluorescent images showing TUNEL positive cells (green); cells are counterstained with DAPI (blue). B: Quantified data from fluorescent images shown in A are graphed as the percent TUNEL positive cells. The small letters (a–d) in image A correspond to the bars shown in graph B below. Significance: ***, p≤0.001.

Development of *Chlamydia pneumoniae* is closely linked to the host cell. Although the intracellular lifestyle is ultimately a lytic process, the ability of this pathogen to actively inhibit cell death through manipulation of apoptotic signaling pathways during development is clearly advantageous to its survival [37], [38]. For example, *Chlamydia pneumoniae* has been shown to protect cells against mitochondria-induced cell death by inhibiting cytochrome c release in response to known apoptosis inducers, and this requires bacterial protein synthesis and the apparent generation of an anti-apoptotic factor in the cytosol of infected cells [39], [40]. We wanted to know if the decreased capacity of the B6.C3H-*sst1* BMDM to support chlamydial development might reflect loss of this anti-apoptotic effect of productive chlamydia infection. We first examined cytotoxicity using propidium iodide staining as a measure of overall cell death. As shown in **Figure 6A**, B6.C3H-*sst1* BMDM had significantly more cytotoxicity following Cp infection at both 48 and 72 hpi compared to wild type B6 BMDM, particularly when subjected to IFN-γ priming. In order to determine if the Cp induced cytotoxicity in the B6.C3H-*sst1* strain was linked to the exaggerated production of IFN-β by infected macrophages, we performed IFN-β neutralization assays. As shown in **Figure 6B**, neutralization of IFN-β, but not IL-10, rescued B6.C3H-*sst1* BMDM from Cp-induced death, regardless of IFN-γ priming, and enhanced the recovery of viable bacteria from infected BMDM (**Figure 6C**). To confirm the mechanism of cell death in the B6.C3H-*sst1* BMDM was due to induction of apoptosis, we repeated these studies using TUNEL staining, which detects DNA fragmentation [41]. We found that Cp infection of IFN-γ primed B6.C3H-*sst1* BMDM consistently led to 10–15% TUNEL positive cells, and that this was significantly inhibited in the presence of neutralizing IFN-β Ab (**Figure 7**). These data demonstrate that the Cp-induced anti-apoptotic program is not effective in blocking cell death in the B6.C3H-*sst1* BMDM, and that this is apparently linked to the exaggerated IFN-β response.

### *Cp* infected B6.C3H-sst1 mice carrying the *sst1* susceptible locus display increased apoptosis in the lungs

**Figure 8:**
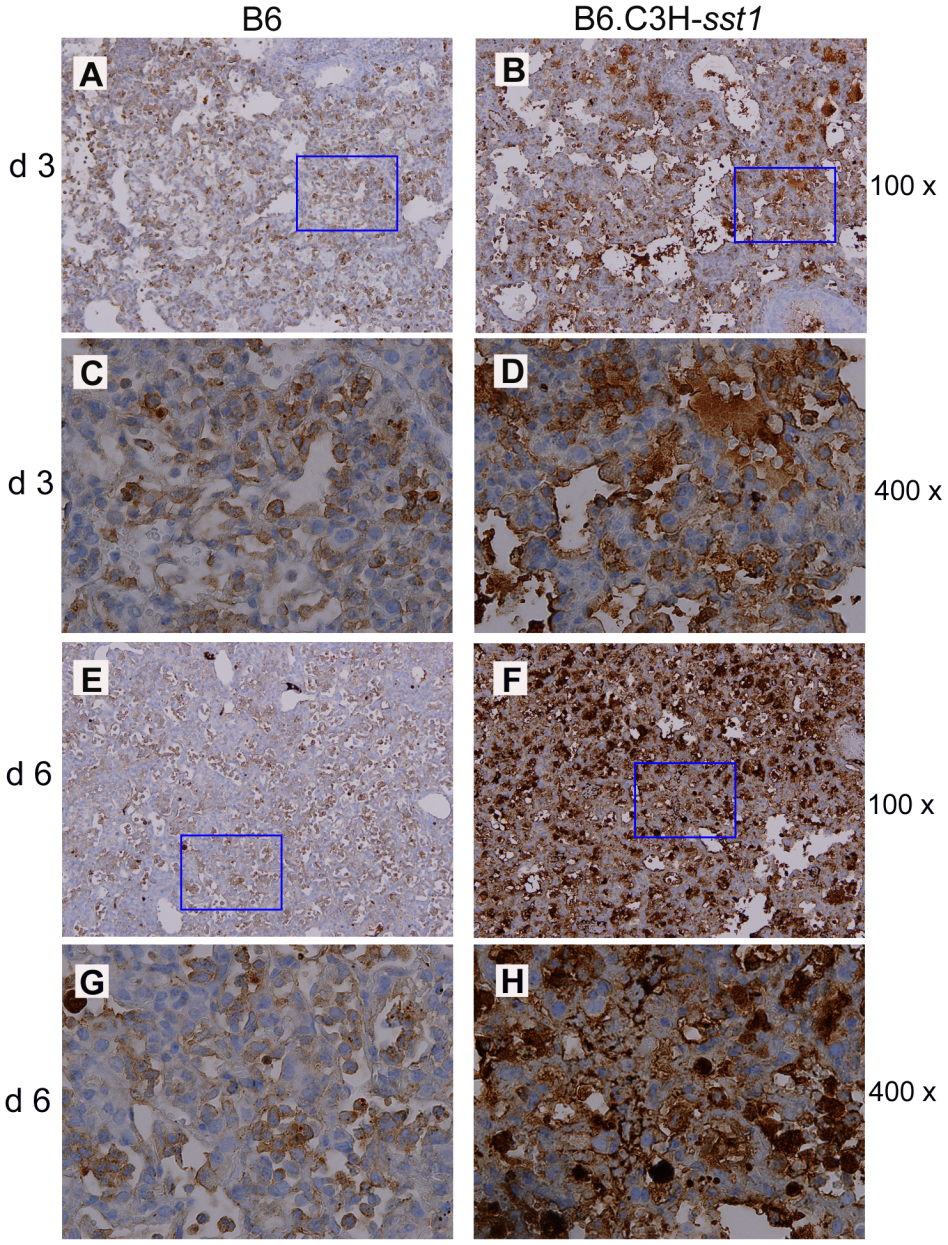
*C. pneumoniae* infected B6.C3H-*sst1* mice show evidence of increased apoptosis in lung tissue. C57BL/6 (B6) or B6.C3H-*sst1* congenic mice were infected with *C. pneumoniae*, and at day 3 and day 6 post infection, lungs were removed for processing, as described in Figure 1. Immunohistochemistry was carried out using monoclonal Ab against cleaved caspase-3. Panels A–B, day 3; E–F, day 6. Panels C–D and G–H are taken from the rectangle area in panels A–B, E–F, respectively. Original magnification: A–B, E–F: 100×; C–D, G–H: 400×. No caspase-3 staining in the lung was observed by immunohistochemistry in mock infected mice (data not shown).

The final question was whether we could link Cp induced cell death and loss of the anti-apoptotic program *in vitro* to the striking *in vivo* phenotype observed in the Cp-infected B6.C3H-*sst1* mice. We examined lungs from infected B6 and B6.C3H-*sst1* mice for evidence of apoptosis, probing for cleaved caspase-3, a known apoptosis effector protein, by immunohistology. As shown in **Figure 8**, we saw a significant increase in the number of cells staining for cleaved caspase-3 by in the B6.C3H-*sst1* mice compared to the B6 control, particularly within the infiltrating neutrophils and macrophages, as well as the endothelial cells lining the blood vessels. The areas of cleaved caspase-3 correlated with the areas of alveolar wound repair, as detected by TTF-1 staining shown in **Figure S2** and suggested an association between enhanced apoptosis and the tissue damage and repair process observed in the lungs of the B6.C3H-*sst1* mice.

## Discussion

*C. pneumoniae* is a common respiratory pathogen, and acute complications are rare except in the extremes of age and severely immunocompromised hosts. Thus, it seems likely that host factors play an important role in determining the outcome of infection. The B6.C3H-*sst1* mouse strain is essentially an immunocompetent host, and differs from the congenic C57BL/6 strain only at a 12-cM interval on mouse chromosome 1 where the *sst1* susceptibility locus has been introgressed from the C3HeB/FeJ inbred mouse strain. This *sst1* locus has primarily been studied as a resistance locus for *M. tuberculosis* [3], [4], [6], [42], where it appears to impart a macrophage-specific phenotype that controls development of lung pathology and bacterial replication.

Like *M. tuberculosis*, respiratory infection with *C. pneumoniae* leads to the development of lung inflammation and granuloma formation to contain the infectious process. The C57BL/6 inbred mouse strain has been widely studied as a model for *C. pneumoniae* infection, and while infected mice display systemic signs of infection, they are largely tolerant of the inoculum size used in our report. We and others have found historically that C57BL/6 mice will clear the infection in the lungs over a 2–3 week period, with complete resolution of lung inflammation without intervention. However, when we examined the response of B6.C3H-*sst1* mice to intranasal infection with *C. pneumoniae*, the impact of the *sst1* susceptibility locus on the progression of disease, in terms of lung pathology and tissue repair, was quite striking, and mice were clinically so impaired that they generally required euthanasia within a week. Histological analysis of the lung tissue from infected *sst1* susceptible B6.C3H-*sst1* mice revealed a significant increase in the influx of neutrophils and macrophages, and marked tissue damage and repair, exemplified by the increased immunostaining for vimentin and TTF-1. Despite this severe clinical course and evidence of significant tissue injury, mice were not apparently impaired in their ability to control the bacterial load, suggesting the outcome was a consequence of the host immune response and not unchecked bacterial replication. This phenotype was quite unlike that reported in known immunocompromised hosts, including mice deficient in MyD88 [43], NOD/RIP2 [44], caspase-1 [45], IL-1β [45], [46], and XIAP [47], where enhanced pathology and worse clinical outcome following *C. pneumoniae* infection could be ascribed to impaired bacterial clearance. Thus, the effect of the *sst1* locus appears to represent an alteration in the host inflammatory response rather than a failure in the host anti-bacterial response.

Because in the tuberculosis and liseriosis models the *sst1* phenotype is primarily a macrophage-specific phenomenon, we conducted a number of studies using bone marrow derived macrophages as an *in vitro* model. While there is some conflicting data with human peripheral blood mononuclear cells [48], *C. pneumoniae* is capable of productively infecting murine macrophages and Shimada *et al.* demonstrated that, in the lungs of *C. pneumoniae* infected mice, the majority of the cells harboring chlamydia were of the macrophage lineage [44]. Thus, these cells are relevant to the pathogenesis of *C. pneumoniae* induced pneumonia, at least in the mouse model. Given that *Chlamydia* species are known to upregulate IFN-γ production during infection, and that macrophages in the lung are likely to be primed during pneumonia due to local cytokine production, we felt a comparison of resting BMDM vs. IFN-γ primed the BMDM would enable us to more closely replicate the lung environment during infection *in vitro*. Similar to what was observed *in vivo*, the *in vitro* data again demonstrated an altered immune response with exaggerated IL-10 and IFN-β secretion observed during infection of primed macrophages. This was unexpected as these two cytokines are not typically upregulated to a significant degree in response to *C. pneumoniae*. Our investigation of the *sst1* locus and the altered cytokine response on chlamydial development provided us with another unique experimental tool with which to study host-pathogen interactions. As chlamydial development is closely tied to the host cell which it parasitizes, the carefully orchestrated cycle of EBs and RBs is largely driven by the intracellular milieu. We observed that inclusion development was clearly impaired, as was the recovery of infectious EBs, in the infected *sst1* susceptible B6.C3H-*sst1* macrophages compared to the wild type C57BL/6 macrophages. This was even more pronounced with IFN-γ primed macrophages. The mechanism by which the IFN-γ primed *sst1* susceptible macrophages fail to support chlamydial development remains unclear, although we did not find any evidence that it was a result of differential IDO expression. It remains to be established whether aberrant maturation of the bacteria within the *sst1* susceptible macrophages reflects their impaired metabolic status and/or possibly altered regulation of autophagy. This link is suggested by a recent finding in the tuberculosis model, where autophagy in infected macrophages was activated by mycobacterial DNA and regulated via the type I interferon pathway [49].

The connection between the *sst1* susceptible locus and activation of the normally silent type I IFN pathway in the context of *C. pneumoniae* infection is most intriguing. Indeed, Byrne and Rothermel had data in 1983 suggestive of the ability of type I IFNs to inhibit growth of some *Chlamydia* species based on studies that used a crude “fibroblast interferon” preparation [50]. We observed that the *sst1* locus regulated the type I IFN response to *C. pneumoniae* infection as IFN-γ primed B6.C3H-*sst1* macrophages secreted large amounts of IFN-β upon *C. pneumoniae* infection while wild type C57BL/6 macrophages did not. Activation of the type I IFN pathway correlated with impaired chlamydial development since IFN-β neutralization improved bacterial recovery. We also found that the *sst1* susceptible locus regulated cell death, both *in vitro* and *in vivo*, with the induction of apoptosis *in vitro* also dependant on the upregulation of IFN-β. Our inhibitor data suggests the mechanism by which the type I IFN pathway is activated by *C. pneumoniae* in the B6.C3H-*sst1* macrophages is dependent on IRF3 and IKK**ξ**/TBK1, and may involve PKR. This, combined with the impaired chlamydial development and fragile-appearing inclusion membranes, suggests to us a model whereby leakage of microbial nucleic acid antigens out of the inclusion might either directly activate cytosolic DNA sensing pathways or target the inclusion to phagosomes that are normally avoided. However, the specific receptors engaged upstream of IRF3 and IKK**ξ**/TBK1 remain undefined, as does the explanation as to why this pathway is not engaged in the IFN-γ primed C57BL/6 macrophages, which carry the *sst1* resistant locus.

The genetic dissection of the *sst1* locus to identify causal genetic variation has been previously performed using a positional cloning approach in the tuberculosis model. These studies revealed complex structure of the candidate region, as it encompasses the largest repeat region in the mouse genome HSR (homogeneously stained region). This repeat was originally described in populations of wild mice, where its maintenance has been attributed to positive selective pressure [51]. We determined that the tuberculosis susceptibility allele was located within the HSR repeat region, which encodes multiple copies of *Ifi75* (interferon-inducible 75) gene (human homologue is known as *Sp110*). One particular variant of *Ifi75* encoding a full length protein corresponding to the Sp110b isoform, which we named *Ipr1* (for ***i***ntracellular ***p***athogen ***r***esistance), was found to be absent in the lungs of *M. tuberculosis* infected *sst1* susceptible mice compared to *sst1* resistant mice, and expression of *Ipr1* in macrophages both *in vitro* and *in vivo* restored many key functions of tuberculosis resistance on the *sst1* susceptible background [6]. However, generation of a single *Ipr1/Sp110* knockout mouse was not feasible due to the repeat structure. Therefore, the specific role of *Ipr1/Sp110* in control of *C. pneumoniae* remains to be explored. Recently the mouse *Sp110* gene has been identified as a regulator of the type I interferon response to cytosolic DNA [52]. Thus while we have no direct data to suggest *Ipr1* is involved in our *C. pneumoniae* model, the similarities of the *sst1* susceptible phenotype in these various infection models with regard to cell death and type I interferon production in macrophages suggests that it is an excellent candidate for future studies. Nevertheless, other polymorphic genes within the *sst1* region may also contribute to the observed differences between the *sst1* susceptible and *sst1* resistant animals. Indeed, clustering of functionally related genes in mammalian genomes, as well as multiple functional polymorphisms within candidate chromosomal loci, has been documented in other forward genetic analyses of inflammatory diseases [53].

Finally, while our data suggest the *sst1* locus regulates a shared defense mechanism against intracellular pathogens, the effects can be quite different amongst these pathogens, likely reflecting differences in their intracellular lifestyles. For example, the *sst1* susceptible mouse strain is clearly compromised in its ability to control the bacterial burden during infection with *M. tuberculosis* and *L. monocytogenes*. In the case of *M. tuberculosis*, we observed unrestricted mycobacterial growth in the *sst1* susceptible mice which correlated with a shift from an apoptotic to a necrotic cell death pathway [3]. Likewise, in the case of *L. monocytogenes*, *sst1* susceptible macrophages were impaired in their ability to kill the bacteria *in vitro* and *in vivo* [5]. Thus, in the context of tuberculosis and listeriosis, the *sst1* susceptible locus appears to also control anti-bacterial resistance.

However, in the case of *C. pneumoniae* we observed a different phenotype. We found no significant difference in the bacterial burden *in vivo* between the *sst1* susceptible and *sst1* resistant mouse strains. In fact, *in vitro*, we found the *sst1* susceptible macrophages were actually impaired in their ability to support bacterial growth and thus bacterial replication was higher in the *sst1* resistant macrophages compared to the *sst1* susceptible macrophages. Yet, the *sst1* susceptible macrophages demonstrated significantly higher IFN-β induction, and *in vivo* the *sst1* susceptible mice displayed more severe inflammation and fibrosis, which correlated with activation of an apoptotic cell death pathway that is normally actively suppressed during infection. This suggests that in the context of *C. pneumoniae* infection the *sst1* susceptible mice are not impaired in their resistance to the infection, but they are impaired in their tolerance to the infection-triggered lung inflammation. That is to say, they develop an inappropriately balanced inflammatory response and, as a result, display more severe disease. This concept of the difference between resistance and tolerance was recently reviewed by Medzhitov *et al.* [54].

We conclude that *sst1* is an important host susceptibility locus which regulates a shared macrophage defense mechanism against diverse intracellular pathogens. Alteration at this locus from a “resistant” allele to a “susceptible” allele results in a severe phenotype in response to respiratory infection with *C. pneumoniae*, which is characterized by arrested bacterial development, activation of the normally silent type I IFN pathway, and induction of apoptosis. The enhanced pathological inflammatory response to the pathogen in this setting demonstrates the important role of host tolerance as a defense mechanism [54], and demonstrates that an effective antibacterial response does not necessarily correlate with an effective immune response. These data could be clinically relevant to acute *C. pneumoniae* infections in the context of a high type I IFN response in the lungs, such as in the setting of a post-viral bacterial pneumonia. Adjunctive therapies to modulate the type I IFN response in the context of intracellular bacteria could improve survival and decrease the morbidity associated with these infections. Finally, further dissection of the *sst1*-mediated pathways using this unique mouse model may identify biomarkers that can be used to predict increased susceptibility to an array of intracellular bacterial infections, and suggest universal therapeutic targets for ameliorating their pathogenesis.

## Materials and Methods

### Ethics statement

All animal use protocols were approved by the Institutional Animal Care and Use Committee (IACUC) of Boston University, approved protocol number AN-14832, in accordance with the recommendations in the Guide for the Care and Use of Laboratory Animals of the National Institutes of Health. Every effort was made to minimize discomfort, pain and distress in the animals. Boston University is accredited by the Association for Assessment and Accreditation of Laboratory Animal Care (AAALAC).

### Reagents

LPS (Escherichia coli serotype O111:B4) was purchased from List Biological Laboratories, INC (Campbell, CA); RPMI1640 was from BioWhittaker® (Lonza Walkersville, MD, USA). Fetal Bovine Serum (FBS; low endotoxin) was from Hyclone (Logan, Utah). TBK1/IKKξ inhibitor BX795 (Cat#: tlrl-bx7), PKR inhibitor 2-Aminopurine (Cat# tlrl-apr), MyD88 inhibitor Pepinh-MYD (Cat# tlrl-pimyd, comes with Pepinh-Control) were purchased from InvivoGen. Anti mouse IDO antibody (clone: mIDO-48) for western blot was from Biolegend; HRP conjugated anti rat IgG was purchased from eBioscience (San Diego, CA, USA). Mouse GAPDH antibody (sc-25778) was purchased from Santa Cruz Biotechnology, Inc. (Santa Cruz, CA, USA).

### Propagation of *Chlamydiae*

*Chlamydia pneumoniae* strain AR39 was obtained from Dr. Li Shen (Louisiana State University, New Orleans, LA). *Chlamydia pneumoniae* was propagated in L929 fibroblasts growing in RPMI-1640 medium supplemented with 10% FBS at 35°C, in a 5% CO2 environment. Following infection, cells were harvested, disrupted by glass beads or sonication (Sonicator 4000, Misonix Sonicators, Newtown, CT, USA), and chlamydiae were separated from cell debris by ultracentrifugation through 32% Renografin-60 (Bracco Diagnostics Inc., Princeton, NJ, USA). Chlamydial EBs were further purified by ultracentrifugation through 40% Renografin-60. After washing twice, the EB pellets were suspended in SPG (sucrose–phosphate–glutamate buffer, pH 7.2) and stored at −80°C. Bacterial titers were calculated as inclusion forming units (IFU) per ml. All the chlamydia stocks used in this study tested negative for *Mycoplasma* contamination by PCR [55]. All experimental procedures involving pathogenic bacteria were carried out with approval from the Institutional Biosafety Committee (IBC) at Boston University Medical Center.

### Mice

C57BL/6 (abbreviated as B6) mice were purchased from Jackson Laboratory. The BL6.C3H-*sst1* transgenic mice were generated from our lab. All animals were housed in groups of 3–5 mice per cage in a controlled environment (temperature 20–22°C, 12∶12 hours light∶dark cycle), given free access to food and water, and maintained under the supervision of veterinary staff from the Laboratory Animal Science Center (LASC) at Boston University Medical Center. All experimental procedures were carried out with approval from the Institutional Animal Care and Use Committee (IACUC) and the Institutional Biosafety Committee (IBC) at Boston University Medical Center.

### Preparation of bone marrow-derived macrophages

Bone marrow derived macrophages (BMDM) were prepared as described previously [46]. Briefly, bone marrows were flushed from femurs and tibiae of the gender and age matched of C57BL/6 or BL6.C3H-*sst1* congenic mice (6–8 wks age) and the cells were cultured in RPMI 1640 supplemented with 10% FBS, 20 µg/ml of gentamicin (Invitrogen, Life Technologies, Grand Island, NY), and 20–30% (v/v) of L929 condition medium (containing M-CSF). The cells were incubated at 37°C, 5% CO_2_ incubator for 7–9 days to allow macrophage differentiation, and removed from gentamicin one day prior to infection with chlamydia.

### Preparation of mouse lung fibroblasts

Mouse lung fibroblasts (MLF) were obtained by culture of lung single-cell suspension prepared from C57BL/6 or BL6.C3H-*sst1* congenic mice. Single-cell suspension was prepared using a gentleMACS Dissociator (MACS Miltenyi Biotec. Auburn, CA). Briefly, lungs were removed from gender and age matched C57BL/6 or BL6.C3H-*sst1* mice and briefly processed using a gentleMACS Dissociator, according to manufacturer's protocol. Lung connective tissue was then digested by incubation with Collagenase D and DNase I for 30 min at 37°C. After second homogenization step, single-cell suspensions were obtained by applying the homogenate to a 70 µm cell strainer filter and the cells were cultured in EMEM supplemented with FBS for 3 weeks. MLF purity was checked by staining for a fibroblast marker using ER-TR7 antibody, and was >90%.

### Murine intranasal infection model

Groups of 6 (mock) or 19–20 (infected) gender and age matched of C57BL/6 and B6.C3H-*sst1* congenic mice (7 to 8 weeks of age) were inoculated with *C. pneumoniae* AR39 or mock via the intranasal route under light anesthesia using ketamine/xylazine mix (60–100/5–10 mg/kg i.p.). Infected mice received 5×10^6^ IFU gradient purified AR39 in 20 µL of SPG followed by 20 µl of PBS; mock infected mice received 20 µl of SPG followed 20 µl PBS. Mice were weighed daily, observed and recorded the signs of distress. At day 3 and day 6, designated mice were euthanized by CO_2_ inhalation. Following collection of the blood by cardiac puncture, the lungs and spleens were removed for cytokines, flow cytometry and bacterial quantification. Lungs and spleens were homogenized in PBS using a Medimachine System (BD Biosciences, San Jose, CA). Three mice from each group were designated for histopathology and immunohistochemistry and processed as follows: lungs were inflated with 10% neutral formalin via the trachea, removed *en bloc* for further formalin fixation, and embedded in paraffin. All *in vivo* experiments were repeated at least twice.

### Clinical score

The clinical score of the infected mice was determined by observing the signs of mouse activity, ruffled fur, hunched posture and degree of the weight loss. Briefly, the mice with healthy, no signs of illness were scored as 0; with subtle ruffled fur, weight loss less than 10% were scored as 1; mice with mild or moderately decreased activity, evident ruffled fur, weight loss within 10–20% were scored as 2; the mice with severe decreased activity and ruffled fur, hunched posture, weight loss over 20%, scored as 3; the mice with no activity, severe hunched posture, scored as 4 (moribund). Both a mean and median clinical score were calculated.

### Quantitative culture of *C. pneumonia*

Quantitative culture of *C. pneumoniae* in lung homogenates, spleen samples, or cell lysates was carried out as described previously [46]. Briefly, for infected tissues, serially diluted samples were inoculated in duplicate onto L929 seeded in a 96-well plate. Infection was initiated by centrifugation at 2000×g for 1 hour at 35°C. Time “zero” for infection was calculated mid-way through the centrifugation step. Infected cells were incubated at 35°/5% CO_2_. After 48–56 hours incubation, the cells were fixed in ice-cold methanol, and inclusions were stained using a *Chlamydia*-specific LPS monoclonal antibody (gift of Dr. You-Xun Zhang, Boston Medical Center), followed by FITC-conjugated secondary antibody; cells were counter stained with Evans blue and DAPI (Sigma, St. Louis, MO). The inclusions were counted under fluorescence microscopy, and calculated as the number of IFU per well/per mouse. For *in vitro* infections of BMDM, cells were disrupted, supernatants collected, and inoculated in duplicate onto L929 fibroblasts seeded in a 96-well plate. At 56–70 hours post inoculation, the cells were fixed and chlamydial inclusion staining was performed as described above. Where indicated, infectivity was quantified from at least 10 images using Image-J software.

### Histopathology and immunohistochemistry

Embedded lung blocks were cut completely in 7–8 µm paraffin sections, and every 10th section stained using routine hematoxylin and eosin (H&E), chloroacetate esterase (CAE) or Masson's trichrome protocols. Immunohistochemistry was carried out on deparaffinized tissue using the following: monoclonal Ab directed against mouse vimentin (clone V9), CD68 (clone KP-1), and Thyroid Transcription Factor-1 (TTF-1) (Clone 8G7G3/1; all from Ventana Medical Systems, Tucson, AZ, USA); polyclonal Ab directed against cleaved caspase-3 (Asp175; Cell Signaling Technology, Danvers, MA, USA); or appropriate isotype controls. Detection was carried out using horseradish peroxidase. Staining for neutrophils and macrophages was quantified by averaging 25 random fields using Image-J software. All slides were reviewed independently by a veterinary pathologist who was blinded as to the experimental design.

### Electron microscopy of *Chlamydia*-infected BMDM

BMDMs from B6 WT or B6.C3H-*sst1* congenic mice were plated onto 6-well plates at 2×10^6^ cells/well overnight, and inoculated with Cp as described in the text. At 69 hpi, the cells were washed twice with cold PBS and detached using a cell lifter. The cells were prefixed with fixative reagent [2.5% glutaraldehyde/2.0% paraformaldehyde in 0.1M sodium cacodylate buffer] at 4°C for overnight. The sample pellets were enrobed and solidified in 2% aqueous agarose (A2576, Sigma) and then dissected into 1 mm cubes. The sample cubes were fixed for 1 h in 1% osmium tetroxide in 0.15 M cacodylate buffer, dehydrated, and embedded in Eponate-Araldite (Ted Pella, Redding, CA, USA). After overnight curing, ultrathin sections were cut and stained with uranyl acetate and lead citrate. The sections were examined at 80 kV in a JEOL transmission electron microscope equipped with a Gatan digital camera.

### Cytokine assay

Supernatants from lung homogenates from infected mice were assayed for cytokines and chemokine using Milliplex®MAP Kit (22 plex, Millipore, Billerica, MA), except for IL-1β for which an individual ELISA was performed. For *in vitro* cytokine assays of infected cells, commercially available ELISA kits were used according to the manufacturer's instructions as follows: IL-1β, IL-6, and IL-10 from R&D Systems (Minneapolis, MN, USA); TNF-α from eBioscience. For mouse IFN-β ELISA, the rat anti-mouse IFN-β mAb (I7662-10A) used as capture antibody was from US Biological (Swampscott, MA, USA); rabbit anti-mouse IFN-β polyclonal Ab (32400-1) used as detection antibody and mouse rIFN-β standard (12400-1), were both from PBL Biomedical Laboratories (Piscataway, NJ, USA); and HRP-conjugated donkey anti-rabbit IgG (711-036-152) was from Jackson ImmunoResearch Laboratories (West Grove, PA, USA). Plates were read in ELx800 Universal Microplate Reader (Bio-Tek, Instruments, Inc., Winooski, VT, USA); and data analyzed using SoftMax Pro 4.6 software. The cytokine induction experiments were performed in triplicate wells, and the data presented as the mean plus or minus SEM.

### mRNA analysis by Reverse Transcriptase (RT)-PCR

Total RNA from 2×10^6^ cells was purified using RNeasy Mini Kit (QIAGEN) following the Manufacturer's instruction. Total RNA (1 µg) was reverse transcribed using the SuperScript® First-Strand Synthesis System from Invitrogen. GoTaq® qPCR Master Mix for Real-Time PCR was purchased from Promega (Madison, WI, USA). The primers used for amplification of IDO were: 5′-ATA TTG CTG TTC CCT ACT GCG A-3′ and 5′-CAT ACA GCA GAC CTT CTG GCA -3′. Cycling condition was 94°C for 2 min (1 cycle); 94°C 30 s, 62°C 30 s, 72°C 1 min for 22 cycles, annealing temperature went down 0.5°C for every next cycle; 94°C 30 s, 52°C 30 s, 72°C 1 min for 30 cycles; 72°C for 7 min (1 cycle). PCR products were visualized using 1.5% agarose gel containing SYBR Safe DNA stain. Relative expression was determined by normalizing data to the housekeeping gene, GAPDH.

### *In vitro* neutralization of IFN-β and IL-10

Where indicated in the text, cells were pretreated with rat anti-mouse INF-β neutralizing antibody (USBiological, Cat# I7662-10A), rat anti-mouse IL-10 neutralizing antibody (eBioscience, Clone: JES5-2A5, Cat#: 16-7102) or rat IgG1 kappa isotype control (Abcam, Cat#: ab18407) for 30 min. Cells were then infected with *C. pneumoniae* as described.

### Cell death assay

For cell survival assay, BMDMs were plated onto 96-well plates at 2.5×10^4^ cells/well with 2% of L929 culture supernatant in triplicate. The cells were pre-treated as described in the text, and infected with *C. pneumoniae* at an MOI of 3∶1. At the designated time points, the cells were stained with Hoechst and propidium iodide for 20 min. Total cell number (Hoechst positive) and dead cell number (propidium iodide positive) were counted by using Cyntellect Celigo cytometer (San Diego, CA). TUNEL staining was performed on BMDMs from BL6.C3H-*sst1* mice. BMDMs were pre-incubated with IFN-γ, isotype control or anti-IFN-β for 6 hr, and then infected with Cp for 10 hr. Apoptotic cells were determined by using Click-iT TUNEL Alexa Flour Imaging Assay (Invitrogen) according to the manufacturer's protocol.

### Statistical analysis

Each experiment was performed 2–3 times to demonstrate the reproducibility of the observed trend. For the *in vivo* studies, mouse number is indicated in the figure or the figure legend. For the *in vitro* studies, each experimental condition was run in triplicate, allowing us to calculate a mean and standard error of the mean. Unless otherwise noted, data was analyzed by calculating the mean and SEM values, and compared using the Student's *t* test (two-tailed). GraphPad Prizm 4.0 (GraphPad, CA) software was used for the analysis. Values of *p*<0.05 were considered significant.

## Supporting Information

Figure S1 **Weight loss and bacterial clearance over time in** ***C. pneumoniae*** **infected B6 vs B6.C3H-** ***sst1*** **mice.** C57BL/6 (B6) or B6.C3H-*sst1* congenic mice were intranasally inoculated with 5×10^6^ IFU *C. pneumoniae* (Cp) or SPG buffer (mock) as described in the Methods. (A) Recorded daily weights of mice over 6 days post infection. Shown above is the weight change relative to the starting weight of each individual mouse. (B) Recovered Cp by quantitative culture from lung homogenates prepared on day 3, 6 and 24 post infection. Data is reported as total IFU Cp per mouse lung. Significance: NS, No significant difference; ***, p≤0.001 Cp infected vs. mock infected. The result is a representative of two independent experiments.
(TIF)

Figure S2 ***C. pneumoniae*** **infected B6.C3H-** ***sst1*** **mice display more evidence of tissue damage and repair compared to B6 mice.** C57BL/6 (B6) or B6.C3H-*sst1* congenic mice were infected with *C. pneumoniae* as described in the Methods. Lungs were removed at the indicated time and processed as follows: immunohistochemistry for detection of vimentin at day 3 (A and B); Masson's trichrome staining at day 6 (C and D); and immunohistochemistry for detection of thyroid transcription factor-1 (TTF-1) at day 6 (E and F). Original magnification: 100×. Shown above are images from one of four infected mice from each genetic background. The result is a representative of two independent experiments.
(TIF)

Figure S3 ***In vitro*** **growth of** ***C.pneumoniae*** **in macrophages vs. fibroblasts.** BMDMs (top panel A) or mouse lung fibroblasts (MLF, bottom panel B) were prepared from C57BL/6 (a–b) or B6.C3H-*sst1* congenic mice (c–d), as described in the Methods. The cells were infected with *C. pneumoniae* at an MOI = 3∶1 (A, BMDM) or 10∶1 (B, MLF), in the absence (a, c) or presence (b,d) of IFN-γ (10 U/ml), in triplicate wells. At 52 hpi, cells were fixed and stained for visualization of Cp inclusions (shown in green), while cells were counter stained with Evans blue (red) and DAPI (blue). Original magnification: 100×. Data is quantified at the far right (e,f) using Image-J software from at least 15 fields of each condition. Graph (e) depicts the mean of cell number from at least 15 images, while graph (f) depicts the percent of cells containing inclusions calculated from at least 20 images. Data is shown as the mean ± SEM. Significance: NS, no significant difference; **, p≤0.01; ***, p≤0.001. Results are representative of 2 (B) or 3 (A) independent experiments.
(TIF)

Figure S4 **Cytokine secretion and** ***C. pneumoniae*** **growth in mouse lung fibroblasts (MLF).** MLF isolated from B6 or B6.C3H-*sst1* mouse were infected with Cp at the indicated MOI (A) or an MOI of 5∶1 (B) in the absence or presence of IFN-γ (10 U/ml). A: Supernatant was harvested at 24 hpi and assayed for IL-6, IL-10 and IFN-β. Shown above is IL-6 data. No detectable IL-10 or IFN-β was observed in the supernatant from Cp infected MLF (not shown). B: Cells were lysed at indicated time points and recovered EBs were quantitatively cultured. Data shown above is representative of 2 (B) or 3 (A) independent experiments.
(TIF)

Table S1 **Clinical scores were determined on day 6 post-infection, and mice were subsequently assigned to two outcome groups based on severity of illness: severe to moribund animals (score 3–4) vs. subtle to moderately ill animals (score 0–2).** Data shown above represents the number of mice that fell into each outcome group. Significance was calculated from the 2×2 contingency table using Fisher's exact t-test (two-tailed). N = 25 mice per genotype, pooled from two independent experiments.
(DOC)

## References

[1] Boyartchuk V, Dietrich W (2002) Genetic dissection of host immune response. Genes Immun 3: 119–122. 12070775 10.1038/sj.gene.6363843

[2] Kramnik I, Boyartchuk V (2002) Immunity to intracellular pathogens as a complex genetic trait. Curr Opin Microbiol 5: 111–117. 11834379 10.1016/s1369-5274(02)00295-3

[3] Kramnik I, Dietrich WF, Demant P, Bloom BR (2000) Genetic control of resistance to experimental infection with virulent Mycobacterium tuberculosis. Proc Natl Acad Sci U S A 97: 8560–8565. 10890913 10.1073/pnas.150227197 PMC26987

[4] Pichugin AV, Yan BS, Sloutsky A, Kobzik L, Kramnik I (2009) Dominant role of the sst1 locus in pathogenesis of necrotizing lung granulomas during chronic tuberculosis infection and reactivation in genetically resistant hosts. Am J Pathol 174: 2190–2201. 19443700 10.2353/ajpath.2009.081075 PMC2684184

[5] Boyartchuk V, Rojas M, Yan BS, Jobe O, Hurt N, et al (2004) The host resistance locus *sst1* controls innate immunity to *Listeria monocytogenes* infection in immunodeficient mice. J Immunol 173: 5112–5120. 15470055 10.4049/jimmunol.173.8.5112

[6] Pan H, Yan BS, Rojas M, Shebzukhov YV, Zhou H, et al (2005) Ipr1 gene mediates innate immunity to tuberculosis. Nature 434: 767–772. 15815631 10.1038/nature03419 PMC1388092

[7] Cliffe ST, Bloch DB, Suryani S, Kamsteeg EJ, Avery DT, et al (2012) Clinical, molecular, and cellular immunologic findings in patients with SP110-associated veno-occlusive disease with immunodeficiency syndrome. J Allergy Clin Immunol 130: 735–742.e6. 22621957 10.1016/j.jaci.2012.02.054

[8] Cliffe ST, Wong M, Taylor PJ, Ruga E, Wilcken B, et al (2007) The first prenatal diagnosis for veno-occlusive disease and immunodeficiency syndrome, an autosomal recessive condition associated with mutations in SP110. Prenat Diagn 27: 674–676. 17510920 10.1002/pd.1759

[9] Abhimanyu, Jha P, Jain A, Arora K, Bose M (2012) Genetic association study suggests a role for SP110 variants in lymph node tuberculosis but not pulmonary tuberculosis in north Indians. Hum Immunol 72: 576–580. 10.1016/j.humimm.2011.03.014 21536091

[10] Babb C, Keet EH, van Helden PD, Hoal EG (2007) SP110 polymorphisms are not associated with pulmonary tuberculosis in a South African population. Hum Genet 121: 521–522. 17287948 10.1007/s00439-007-0335-1

[11] Tosh K, Campbell SJ, Fielding K, Sillah J, Bah B, et al (2006) Variants in the SP110 gene are associated with genetic susceptibility to tuberculosis in West Africa. Proc Natl Acad Sci U S A 103: 10364–10368. 16803959 10.1073/pnas.0603340103 PMC1502463

[12] Cook PJ, Davies P, Tunnicliffe W, Ayres JG, Honeybourne D, et al (1998) Chlamydia pneumoniae and asthma. Thorax 53: 254–259. 9741366 10.1136/thx.53.4.254 PMC1745183

[13] Emre U, Roblin PM, Gelling M, Dumornay W, Rao M, et al (1994) The association of Chlamydia pneumoniae infection and reactive airway disease in children. Arch Pediatr Adolesc Med 148: 727–732. 8019629 10.1001/archpedi.1994.02170070065013

[14] Baumert J, Schmidt KH, Eitner A, Straube E, Rodel J (2009) Host cell cytokines induced by Chlamydia pneumoniae decrease the expression of interstitial collagens and fibronectin in fibroblasts. Infect Immun 77: 867–876. 19047405 10.1128/IAI.00566-08 PMC2632053

[15] Campbell LA, Kuo CC (2004) Chlamydia pneumoniae–an infectious risk factor for atherosclerosis? Nat Rev Microbiol 2: 23–32. 15035006 10.1038/nrmicro796

[16] Borel N, Pospischil A, Dowling RD, Dumrese C, Gaydos CA, et al (2012) Antigens of persistent Chlamydia pneumoniae within coronary atheroma from patients undergoing heart transplantation. J Clin Pathol 65: 171–177. 22049224 10.1136/jclinpath-2011-200270

[17] Borel N, Summersgill JT, Mukhopadhyay S, Miller RD, Ramirez JA, et al (2008) Evidence for persistent Chlamydia pneumoniae infection of human coronary atheromas. Atherosclerosis 199: 154–161. 18028932 10.1016/j.atherosclerosis.2007.09.026

[18] Timms P, Good D, Wan C, Theodoropoulos C, Mukhopadhyay S, et al (2009) Differential transcriptional responses between the interferon-gamma-induction and iron-limitation models of persistence for Chlamydia pneumoniae. J Microbiol Immunol Infect 42: 27–37. 19424556

[19] Matsumoto A, Manire GP (1970) Electron microscopic observations on the effects of penicillin on the morphology of Chlamydia psittaci. J Bacteriol 101: 278–285. 5413965 10.1128/jb.101.1.278-285.1970 PMC250478

[20] Coles AM, Reynolds DJ, Harper A, Devitt A, Pearce JH (1993) Low-nutrient induction of abnormal chlamydial development: a novel component of chlamydial pathogenesis? FEMS Microbiol Lett 106: 193–200. 8454184 10.1111/j.1574-6968.1993.tb05958.x

[21] Raulston JE (1997) Response of Chlamydia trachomatis serovar E to iron restriction in vitro and evidence for iron-regulated chlamydial proteins. Infect Immun 65: 4539–4547. 9353031 10.1128/iai.65.11.4539-4547.1997 PMC175652

[22] Pantoja LG, Miller RD, Ramirez JA, Molestina RE, Summersgill JT (2001) Characterization of Chlamydia pneumoniae persistence in HEp-2 cells treated with gamma interferon. Infect Immun 69: 7927–7932. 11705979 10.1128/IAI.69.12.7927-7932.2001 PMC98893

[23] Yan BS, Kirby A, Shebzukhov YV, Daly MJ, Kramnik I (2006) Genetic architecture of tuberculosis resistance in a mouse model of infection. Genes Immun 7: 201–210. 16452998 10.1038/sj.gene.6364288

[24] Xing Z, Gauldie J, Cox G, Baumann H, Jordana M, et al (1998) IL-6 is an antiinflammatory cytokine required for controlling local or systemic acute inflammatory responses. J Clin Invest 101: 311–320. 9435302 10.1172/JCI1368 PMC508569

[25] Benveniste EN, Qin H (2007) Type I interferons as anti-inflammatory mediators. Sci STKE 2007: pe70. 18073382 10.1126/stke.4162007pe70

[26] Billiau A (2006) Anti-inflammatory properties of Type I interferons. Antiviral Res 71: 108–116. 16626815 10.1016/j.antiviral.2006.03.006 PMC7114336

[27] Guarda G, Braun M, Staehli F, Tardivel A, Mattmann C, et al (2011) Type I interferon inhibits interleukin-1 production and inflammasome activation. Immunity 34: 213–223. 21349431 10.1016/j.immuni.2011.02.006

[28] Chang EY, Guo B, Doyle SE, Cheng G (2007) Cutting edge: involvement of the type I IFN production and signaling pathway in lipopolysaccharide-induced IL-10 production. J Immunol 178: 6705–6709. 17513714 10.4049/jimmunol.178.11.6705

[29] Peters RT, Liao SM, Maniatis T (2000) IKKepsilon is part of a novel PMA-inducible IkappaB kinase complex. Mol Cell 5: 513–522. 10882136 10.1016/s1097-2765(00)80445-1

[30] Shimada T, Kawai T, Takeda K, Matsumoto M, Inoue J, et al (1999) IKK-i, a novel lipopolysaccharide-inducible kinase that is related to IkappaB kinases. Int Immunol 11: 1357–1362. 10421793 10.1093/intimm/11.8.1357

[31] Bonnard M, Mirtsos C, Suzuki S, Graham K, Huang J, et al (2000) Deficiency of T2K leads to apoptotic liver degeneration and impaired NF-kappaB-dependent gene transcription. Embo J 19: 4976–4985. 10990461 10.1093/emboj/19.18.4976 PMC314216

[32] Tojima Y, Fujimoto A, Delhase M, Chen Y, Hatakeyama S, et al (2000) NAK is an IkappaB kinase-activating kinase. Nature 404: 778–782. 10783893 10.1038/35008109

[33] Fitzgerald KA, McWhirter SM, Faia KL, Rowe DC, Latz E, et al (2003) IKKepsilon and TBK1 are essential components of the IRF3 signaling pathway. Nat Immunol 4: 491–496. 12692549 10.1038/ni921

[34] Clark K, Plater L, Peggie M, Cohen P (2009) Use of the pharmacological inhibitor BX795 to study the regulation and physiological roles of TBK1 and IkappaB kinase epsilon: a distinct upstream kinase mediates Ser-172 phosphorylation and activation. J Biol Chem 284: 14136–14146. 19307177 10.1074/jbc.M109.000414 PMC2682862

[35] Kaufman RJ (1999) Double-stranded RNA-activated protein kinase mediates virus-induced apoptosis: a new role for an old actor. Proc Natl Acad Sci U S A 96: 11693–11695. 10518510 10.1073/pnas.96.21.11693 PMC33789

[36] Sugiyama T, Gotou T, Moriyama K, Kajiura N, Hasegawa T, et al (2012) Mechanism of inhibition of lipopolysaccharide-induced interferon-beta production by 2-aminopurine. Mol Immunol 52: 299–304. 22750230 10.1016/j.molimm.2012.06.008

[37] Fischer SF, Schwarz C, Vier J, Hacker G (2001) Characterization of antiapoptotic activities of Chlamydia pneumoniae in human cells. Infect Immun 69: 7121–7129. 11598088 10.1128/IAI.69.11.7121-7129.2001 PMC100101

[38] Wahl C, Oswald F, Simnacher U, Weiss S, Marre R, et al (2001) Survival of Chlamydia pneumoniae-infected Mono Mac 6 cells is dependent on NF-kappaB binding activity. Infect Immun 69: 7039–7045. 11598079 10.1128/IAI.69.11.7039-7045.2001 PMC100084

[39] Fan T, Lu H, Hu H, Shi L, McClarty GA, et al (1998) Inhibition of apoptosis in chlamydia-infected cells: blockade of mitochondrial cytochrome c release and caspase activation. J Exp Med 187: 487–496. 9463399 10.1084/jem.187.4.487 PMC2212145

[40] Fischer SF, Hacker G (2003) Characterization of antiapoptotic activities of Chlamydia pneumoniae in infected cells. Ann N Y Acad Sci 1010: 565–567. 15033792 10.1196/annals.1299.105

[41] Gavrieli Y, Sherman Y, Ben-Sasson SA (1992) Identification of programmed cell death in situ via specific labeling of nuclear DNA fragmentation. J Cell Biol 119: 493–501. 1400587 10.1083/jcb.119.3.493 PMC2289665

[42] Kramnik I (2008) Genetic Dissection of Host Resistance to Mycobacterium tuberculosis : The *sst1* Locus and the *Ipr1* Gene. In: Beutler B, editor. Immunology, Phenotype First: How Mutations Have Established New Principles and Pathways in Immunology Berlin Heidelberg: Springer-Verlag pp. 124–148. 10.1007/978-3-540-75203-5_6 18727490

[43] Naiki Y, Michelsen KS, Schroder NW, Alsabeh R, Slepenkin A, et al (2005) MyD88 is pivotal for the early inflammatory response and subsequent bacterial clearance and survival in a mouse model of Chlamydia pneumoniae pneumonia. J Biol Chem 280: 29242–29249. 15964841 10.1074/jbc.M503225200

[44] Shimada K, Chen S, Dempsey PW, Sorrentino R, Alsabeh R, et al (2009) The NOD/RIP2 pathway is essential for host defenses against Chlamydophila pneumoniae lung infection. PLoS Pathog 5: e1000379. 19360122 10.1371/journal.ppat.1000379 PMC2660273

[45] Shimada K, Crother TR, Karlin J, Chen S, Chiba N, et al (2011) Caspase-1 dependent IL-1beta secretion is critical for host defense in a mouse model of Chlamydia pneumoniae lung infection. PLoS One 6: e21477. 21731762 10.1371/journal.pone.0021477 PMC3121765

[46] He X, Mekasha S, Mavrogiorgos N, Fitzgerald KA, Lien E, et al (2010) Inflammation and fibrosis during Chlamydia pneumoniae infection is regulated by IL-1 and the NLRP3/ASC inflammasome. J Immunol 184: 5743–5754. 20393140 10.4049/jimmunol.0903937 PMC3156096

[47] Prakash H, Albrecht M, Becker D, Kuhlmann T, Rudel T (2010) Deficiency of XIAP leads to sensitization for Chlamydophila pneumoniae pulmonary infection and dysregulation of innate immune response in mice. J Biol Chem 285: 20291–20302. 20427267 10.1074/jbc.M109.096297 PMC2888442

[48] Wolf K, Fischer E, Hackstadt T (2005) Degradation of Chlamydia pneumoniae by peripheral blood monocytic cells. Infect Immun 73: 4560–4570. 16040967 10.1128/IAI.73.8.4560-4570.2005 PMC1201216

[49] Watson RO, Manzanillo PS, Cox JS (2012) Extracellular M. tuberculosis DNA targets bacteria for autophagy by activating the host DNA-sensing pathway. Cell 150: 803–815. 22901810 10.1016/j.cell.2012.06.040 PMC3708656

[50] Byrne GI, Rothermel CD (1983) Differential susceptibility of chlamydiae to exogenous fibroblast interferon. Infect Immun 39: 1004–1005. 6832807 10.1128/iai.39.2.1004-1005.1983 PMC348050

[51] Agulnik S, Plass C, Traut W, Winking H (1993) Evolution of a long-range repeat family in chromosome 1 of the genus Mus. Mamm Genome 4: 704–710. 7906569 10.1007/BF00357793

[52] Lee MN, Roy M, Ong SE, Mertins P, Villani AC, et al (2013) Identification of regulators of the innate immune response to cytosolic DNA and retroviral infection by an integrative approach. Nat Immunol 14: 179–185. 23263557 10.1038/ni.2509 PMC3838897

[53] Limaye N, Belobrajdic KA, Wandstrat AE, Bonhomme F, Edwards SV, et al (2008) Prevalence and evolutionary origins of autoimmune susceptibility alleles in natural mouse populations. Genes Immun 9: 61–68. 18094711 10.1038/sj.gene.6364446

[54] Medzhitov R, Schneider DS, Soares MP (2012) Disease tolerance as a defense strategy. Science 335: 936–941. 22363001 10.1126/science.1214935 PMC3564547

[55] Ossewaarde JM, de Vries A, Bestebroer T, Angulo AF (1996) Application of a Mycoplasma group-specific PCR for monitoring decontamination of Mycoplasma-infected Chlamydia sp. strains. Appl Environ Microbiol 62: 328–331. 8593037 10.1128/aem.62.2.328-331.1996 PMC167802

