# Supplementary figures and images for: The sst1 Resistance Locus Regulates Evasion of Type I Interferon Signaling by Chlamydia pneumoniae as a Disease Tolerance Mechanism
Source: PLoS Pathog. 2013 Aug 29;9(8):e1003569. doi: 10.1371/journal.ppat.1003569 (PMC3757055; doi:10.1371/journal.ppat.1003569)

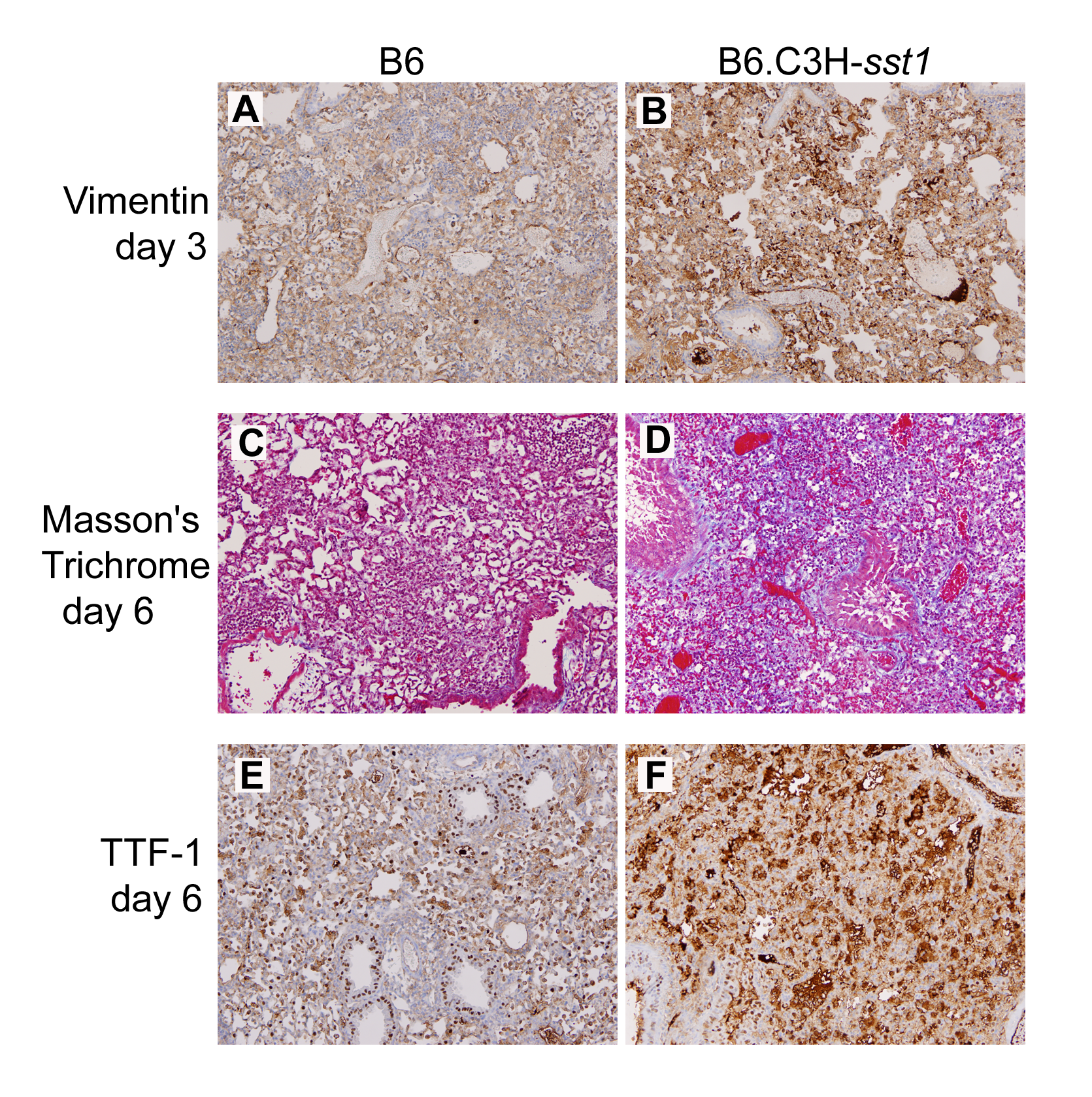

Supplement: Figure S2 — C. pneumoniae infected B6.C3H- sst1 mice display more evidence of tissue damage and repair compared to B6 mice. C57BL/6 (B6) or B6.C3H-sst1 congenic mice were infected with C. pneumoniae as described in the Methods. Lungs were removed at the indicated time and processed as follows: immunohistochemistry for detection of vimentin at day 3 (A and B); Masson's trichrome staining at day 6 (C and D); and immunohistochemistry for detection of thyroid transcription factor-1 (TTF-1) at day 6 (E and F). Original magnification: 100×. Shown above are images from one of four infected mice from each genetic background. The result is a representative of two independent experiments. (TIF) [file ppat.1003569.s002.tif]

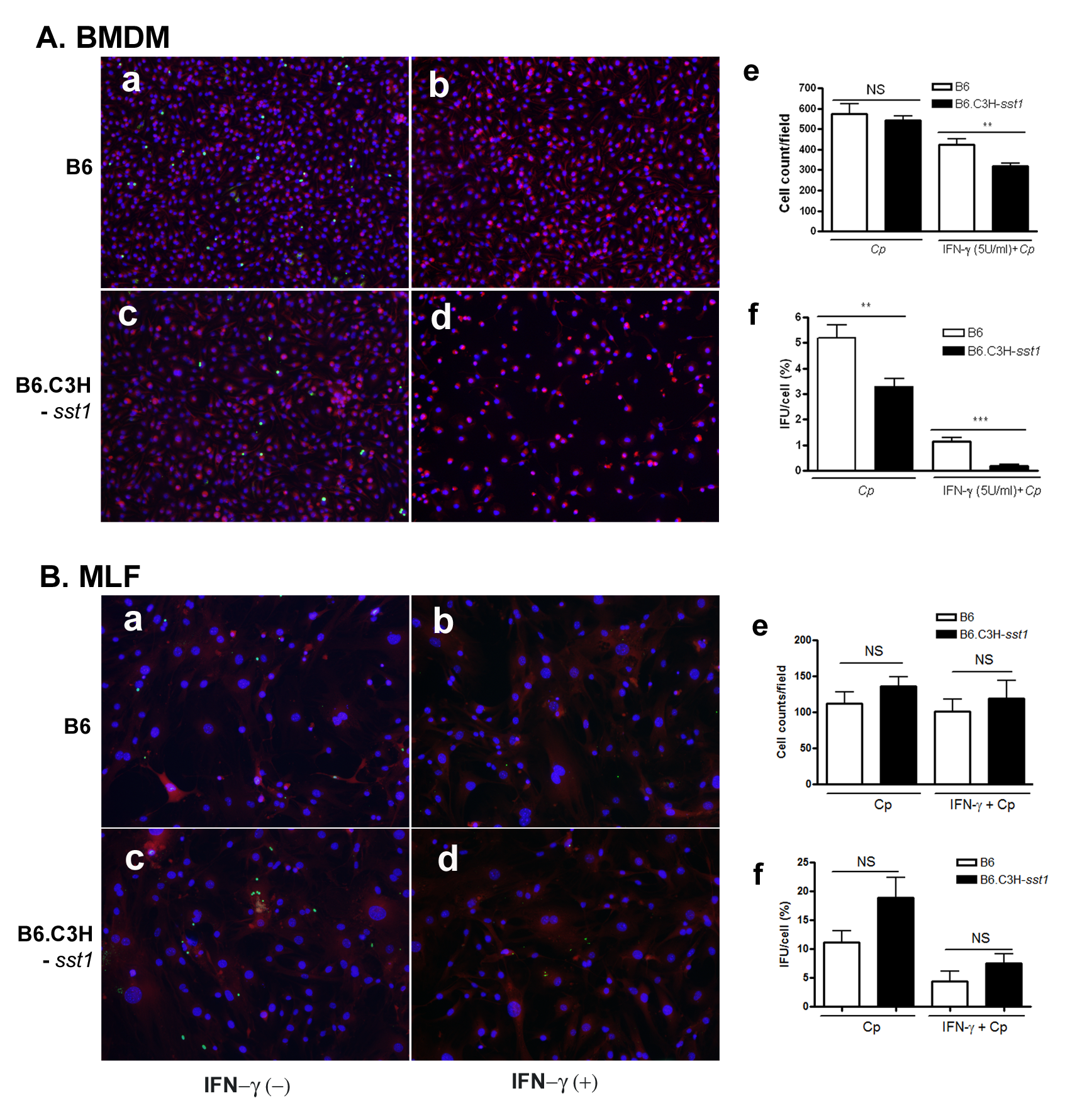

Supplement: Figure S3 — In vitro growth of C.pneumoniae in macrophages vs. fibroblasts. BMDMs (top panel A) or mouse lung fibroblasts (MLF, bottom panel B) were prepared from C57BL/6 (a–b) or B6.C3H-sst1 congenic mice (c–d), as described in the Methods. The cells were infected with C. pneumoniae at an MOI = 3∶1 (A, BMDM) or 10∶1 (B, MLF), in the absence (a, c) or presence (b,d) of IFN-γ (10 U/ml), in triplicate wells. At 52 hpi, cells were fixed and stained for visualization of Cp inclusions (shown in green), while cells were counter stained with Evans blue (red) and DAPI (blue). Original magnification: 100×. Data is quantified at the far right (e,f) using Image-J software from at least 15 fields of each condition. Graph (e) depicts the mean of cell number from at least 15 images, while graph (f) depicts the percent of cells containing inclusions calculated from at least 20 images. Data is shown as the mean ± SEM. Significance: NS, no significant difference; **, p≤0.01; ***, p≤0.001. Results are representative of 2 (B) or 3 (A) independent experiments. (TIF) [file ppat.1003569.s003.tif]
