# Supplementary material for: The sst1 Resistance Locus Regulates Evasion of Type I Interferon Signaling by Chlamydia pneumoniae as a Disease Tolerance Mechanism
Source: PLoS Pathog. 2013 Aug 29;9(8):e1003569. doi: 10.1371/journal.ppat.1003569 (PMC3757055; doi:10.1371/journal.ppat.1003569)
Supplement: Table S1 — Clinical scores were determined on day 6 post-infection, and mice were subsequently assigned to two outcome groups based on severity of illness: severe to moribund animals (score 3–4) vs. subtle to moderately ill animals (score 0–2). Data shown above represents the number of mice that fell into each outcome group. Significance was calculated from the 2×2 contingency table using Fisher's exact t-test (two-tailed). N = 25 mice per genotype, pooled from two independent experiments. (DOC) [file ppat.1003569.s005.doc]

**Table S1: 2x2 Contingency table of clinical scores**

| **Outcome** | **Severe to Moribund**  (score 3-4) | | **Subtle to moderate**  (score 0-2) | Total |
| --- | --- | --- | --- | --- |
| **B6** | 5 | | 20 | 25 |
| **B6.C3H-*sst1*** | 15 | | 10 | 25 |
| Total | 20 | | 30 | 50 |
|  |  | Fisher’s exact *t*-test (two-tailed) | | p = 0.0086 |
